# Unveiling the role of base catalysts in the thiol-Michael addition reaction in liquid crystal oligomers and liquid crystal elastomers†

**DOI:** 10.1039/d6sc03222b

**Published:** 2026-05-13

**Authors:** Ramazan Umut Dinc, Johan Lub, Xianwen Lou, Augustinus J. J. Kragt, Albert P. H. J. Schenning

**Affiliations:** a Laboratory of Stimuli-responsive Functional Materials and Devices (SFD), Department of Chemical Engineering and Chemistry, Eindhoven University of Technology P.O. Box 513 5600 MB Eindhoven The Netherlands a.p.h.j.schenning@tue.nl

## Abstract

Liquid crystal oligomers (LCOs) and liquid crystal elastomers (LCEs) are currently receiving much attention as stimuli-responsive polymer materials capable of adapting to environmental changes such as light and temperature, resulting in changes in functional properties including shape and colour. The base-catalysed thiol–Michael addition is widely used to synthesize LCOs and LCEs, yet the role of the catalyst is often overlooked. This study shows that commonly used amine catalysts form base adducts with the liquid crystal building blocks. In the case of triethylamine, this side reaction is avoided, enabling a 95% acrylate conversion in the thiol-Michael addition reaction used to synthesize LCOs. Thermomechanical analyses of the corresponding LCEs demonstrate that the materials free from nucleophilic base adducts and with a high thiol-Michael addition reaction conversion exhibit an increased Young's modulus and enhanced stiffness and stress resistance, which is likely attributable to a higher crosslink density. Our work provides guidelines for the synthesis of liquid crystal oligomers and elastomers, potentially improving the performance of stimuli-responsive materials and devices.

## Abstract 2

Our studies unveil the role of base catalysts in the thiol-Michael addition reaction in liquid crystal oligomers and liquid crystal elastomers providing a pathway for improving the performance of stimuli-responsive materials and devices.

## Graphical abstract

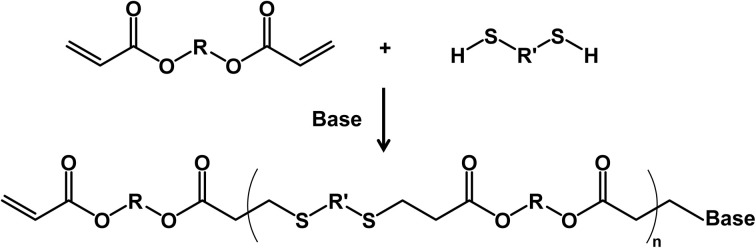

## Introduction

The thiol-Michael addition reaction is a widely used “click-chemistry” type reaction in polymer chemistry, employed to synthesize and modify polymer materials.^1^ In this reaction, a thiol group adds to a Michael acceptor, such as acrylates in the presence of a catalyst. The reaction is widely regarded as highly selective and versatile; operating under mild reaction conditions while affording good yields. Two mechanisms have been proposed in the literature for the thiol-Michael addition: the base-catalysed and the nucleophile-initiated mechanism. In the base-catalysed mechanism, the catalyst acts as a base and deprotonates the thiol to form a thiolate in the initiation step. The thiolate then adds to the acrylate vinyl group. Subsequently, proton donation of the protonated base takes place, and the catalyst continues the next cycles. In the nucleophile-initiated mechanism, the catalyst acts as a nucleophile and adds itself to the vinyl group of the acrylate for the initiation. A thiol then donates a proton to this negatively charged complex becoming a thiolate, after which the reaction propagates similar to the base-catalysed mechanism.^2^ However, the nucleophile-initiated pathway results in the loss of the catalyst for the subsequent cycles and gives a conjugated side product. It has been reported in the literature that the choice of the catalyst, the type of vinyl containing group, and the solvent highly influence the addition mechanism.^3^ Therefore, due to their basic properties, amine catalysts have been widely used to facilitate thiol-Michael additions under mild and clean conditions^4^ assuming that they only follow the base-catalysed mechanism.

Liquid crystal oligomers (LCOs) and liquid crystal elastomers (LCEs) are currently receiving much attention as ‘smart’ polymer materials capable of adapting to environmental changes such as light and temperature, resulting in changes in functional properties including shape and colour.^5–13^ The base-catalysed thiol-Michael addition reaction is commonly used to fabricate these programmable shape and colour changing polymer materials.^14–18^ Typical catalysts include dipropylamine (DPA), α-methyl benzylamine (α-MBA), 1,8-diazabicyclo[5.4.0]undec-7-ene (DBU), and triethylamine (TEA). One way of synthesizing main chain LCOs is *via* thiol-Michael addition of a diacrylate liquid crystal and a dithiol, catalysed by an amine as the base. An optical response is often achieved by incorporating a chiral dopant, which induces a chiral nematic phase and structural colour. In the case of LCEs, shape changes are generated upon undergoing the order-to-disorder phase transition, *i.e.* the nematic-isotropic phase transition temperature (*T*_NI_). This phase transition can be programmed by controlling the crosslink density and the nature of the liquid crystal building blocks within the LCEs. The general procedure begins with the base catalysed thiol-Michael addition of a diacrylate liquid crystal and thiol derivatives as crosslinkers. Subsequently, the slightly crosslinked polymer is stretched, and the remaining free acrylates are photopolymerized by UV light exposure to produce the final LCE smart material.^19,20^ Due to the sequential photopolymerization step, the role of the catalyst has not been extensively explored.^21–24^ However, potential side reactions during the thiol-Michael addition reaction may influence the material's responsive and optical properties, lifetime, and fatigue behaviour.

**Fig. 1:**
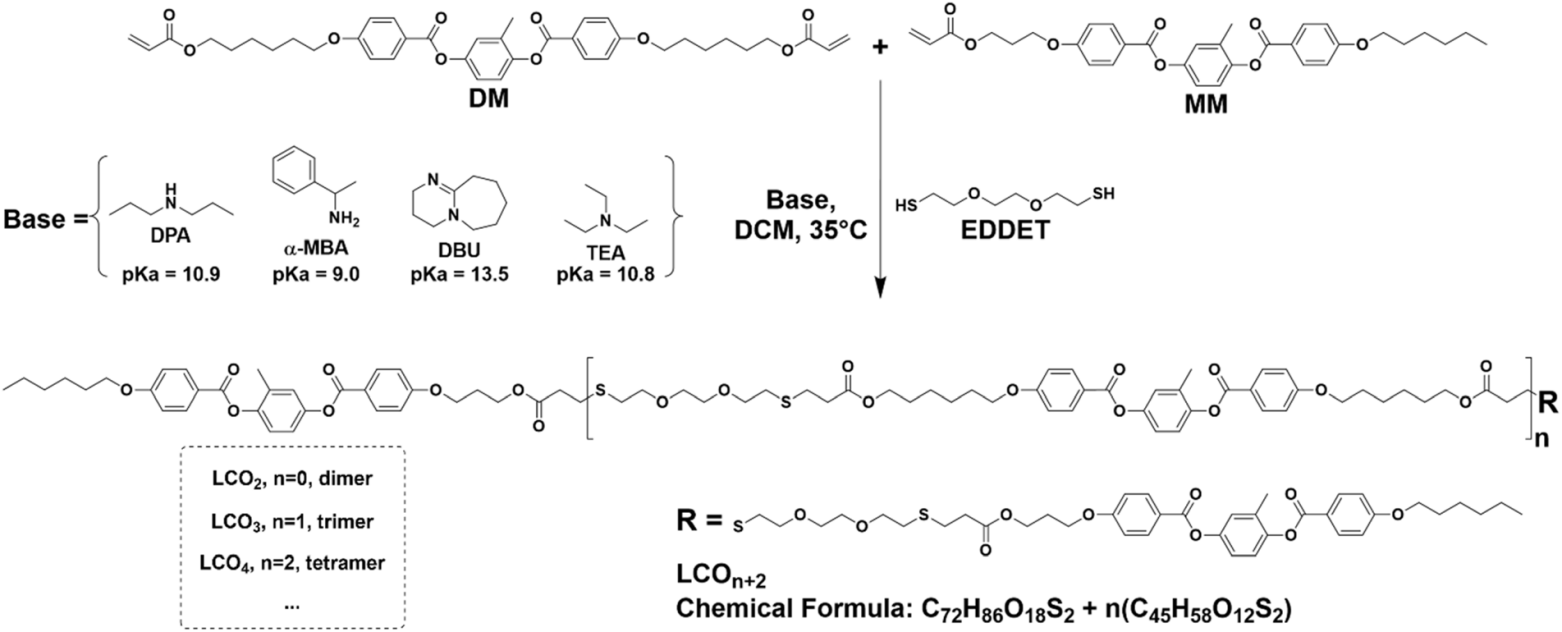
Schematic of the model reaction to prepare LCOs where dichloromethane (DCM) is the solvent.

In this work, we unveil the role of four frequently used amine-based catalysts in the thiol-Michael addition reaction in LCOs and LCEs. We evaluated the catalytic performance of these catalysts in a LC oligomerization reaction *via* thiol-Michael addition. The model reaction involves a diacrylate mesogen (DM), a monoacrylate mesogen (MM) and a dithiol linker (EDDET), resulting in end-capped LCOs (Fig. 1). For the three catalysts, nucleophilic base–acrylate adducts were identified as side products by MALDI-ToF-MS. In contrast, TEA as the base did not show nucleophilic addition, and by adjusting the reaction concentration a 95% yield was achieved.

## Results and discussion

**Fig. 2:**
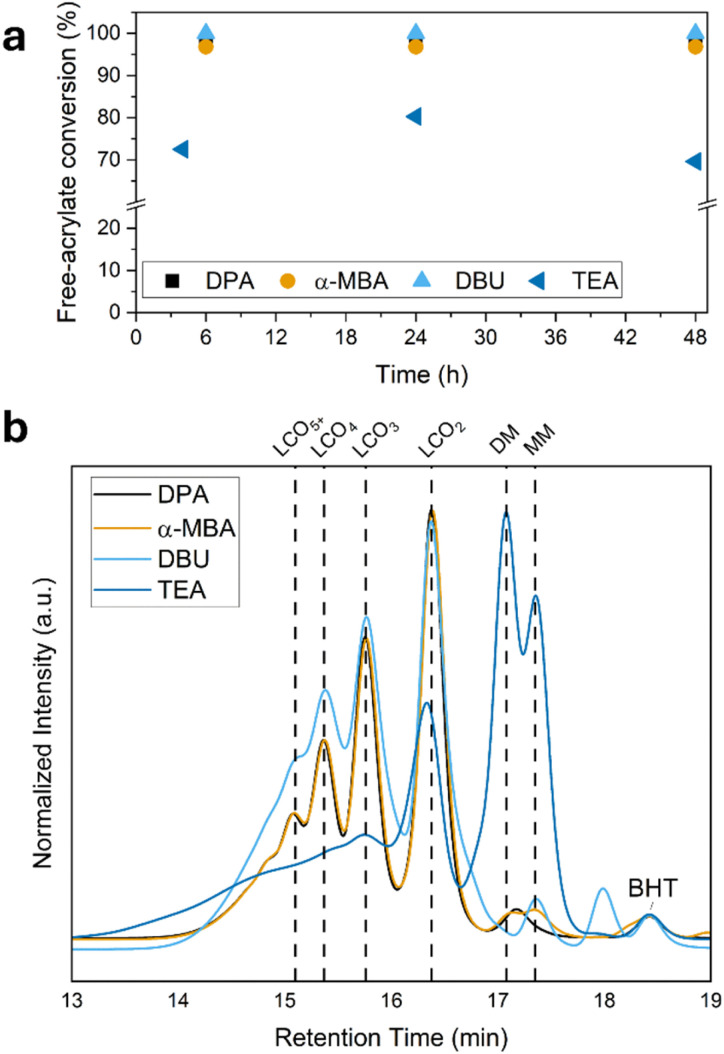
(a) Acrylate conversion calculated from the ^1^H-NMR data as a function of time for the selected catalysts. (b) GPC profiles of the molecular distribution of the syntheses after 48 hour and acid–base treatment. “LCO_2+*n*_” represents the oligomer size at which monomer units linked with dithiols. BHT stands for butylated hydroxytoluene in the solvent.

We have investigated the role of four commonly used bases, DPA, α-MBA, DBU, and TEA (Fig. 1), in the thiol-Michael addition reaction. These bases which contain a tertiary, secondary, or primary amine have high and comparable pKa values, with DBU exhibiting the highest basicity. We aimed to synthesize endcapped LCOs to simplify the analysis. In a typical reaction, 1 eq. of DM, 2 eq. of EDDET and 2 eq. of MM were reacted in the presence of a base in dichloromethane (DCM), at 35 °C (Fig. 1) in a closed vial under an argon atmosphere. The total concentration of the reactants was maintained at approximately 0.23 M, with a catalyst concentration of 14 mM consistent with typical reaction conditions reported for LCOs and LCEs.^14–18^ The reactions were monitored for up to 48 h. Remaining free acrylates in the mixture were calculated by analysing ^1^H-NMR spectra.^25^ It was observed that DPA, α-MBA and DBU achieved nearly 100% acrylate conversion within 6 h, with no significant increase in conversion upon further reaction up to 48 h. In contrast, the reaction employing TEA as the base reached a plateau of 80% acrylate conversion after 24 h (Fig. 2a). Remarkably, the conversion decreased to 70% at 48 h, which might indicate an equilibrium reaction. After 48 hours, the reactions were further analysed by GPC. In the GPC profiles (Fig. 2b), individual peaks corresponding to oligomers of varying chain lengths are visible and show a range of molecular weight distributions. Oligomers containing one to four mesogenic units are clearly distinguishable.^26^ For the ease of terminology, LCO_2_, LCO_3_, and LCO_4_ are hereafter referred to as dimer, trimer, and tetramer, respectively (Fig. 1). The profiles for DPA (Fig. S6) and α-MBA (Fig. S13) catalysed reactions appear similar and exhibit comparable polydispersity indices (PDI) of 1.51 and 1.55 while DBU (Fig. S20) and TEA (Fig. S27) showed increased PDIs of 1.81 and 3.52, respectively. In the case of DBU, the GPC profile contains an additional distinguished peak at 18.0 min, suggesting the presence of species with a lower molecular weight than MM and DM, which may indicate a cleaving side reaction (*vide infra*). The least effective catalyst, TEA, shows a relatively high area percentage of monomers, which is consistent with the lower conversion compared to the other catalysts. However, it is also noticeable that the TEA catalysed reaction leads to the formation of higher molecular weight species compared to those obtained with the other bases (Fig. 2b and S27). Notably, the monomer peaks in the GPC profiles account for more than 3% of the total area in the case of DPA, α-MBA and DBU. These values do not correlate with the remaining free acrylates determined by ^1^H-NMR (<3%), suggesting the presence of possible side products that do not contain free acrylate moieties.

**Fig. 3:**
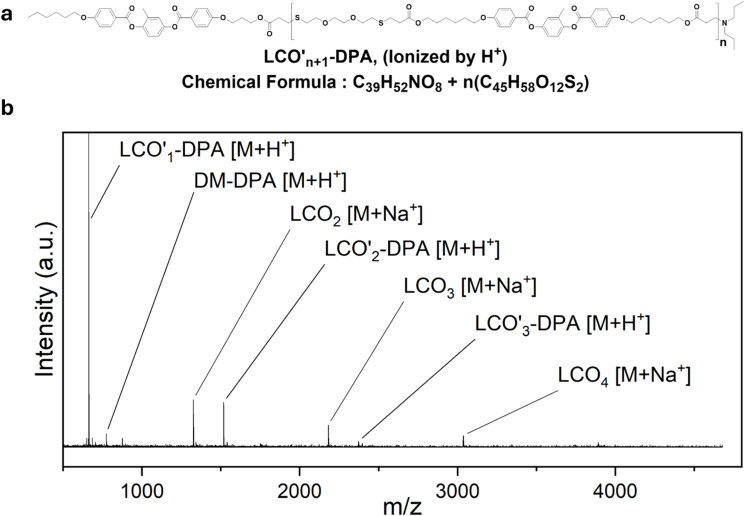
(a) The molecular structure of the DPA reacted base adduct. (b) The MALDI-ToF-MS analysis of the DPA catalysed thiol-Michael addition, showing DPA reacted base adducts alongside typical LCO *m*/*z* values.

To investigate the discrepancy between the ^1^H-NMR and GPC results, the samples were further analysed using MALDI-ToF-MS (Fig. 3). In the case of the DPA catalysed sample, the analysis revealed no unreacted monomer signals. Instead, the presence of dimers, trimers and tetramers was observed (Fig. 3b) consistent with the GPC results. Remarkably, additional low-intensity peaks indicate base reacted LCO species. The presence of DPA adducted dimers, trimers and tetramers suggests a nucleophilic addition of the base (Fig. 3a) during the thiol-Michael reaction. Similarly, MALDI-ToF-MS analyses of the α-MBA and DBU catalysed samples revealed the presence of base reacted LCOs, alongside the expected LCO species (Fig. S15 and S22). Additionally, in the DBU catalysed reaction, a trace of a cleaved DM product was found, likely arising from a reaction of DBU with the sequential aromatic ester linkage of the mesogenic groups. The presence of this cleavage product supports the low molecular weight peak at 18.0 min in the corresponding GPC profile of the same reaction (Fig. 2b and S21). Finally, in the TEA catalysed sample, the MALDI-ToF-MS analysis revealed only the expected LCOs with no evidence of nucleophilic addition to the acrylate groups. The absence of TEA adducts may be attributed to steric hindrance associated with the tertiary amine structure of TEA. Interestingly, traces of intermediate molecules with thiol-end groups were detected together with signals corresponding to free DM and MM (Fig. S29) which is consistent again with the lower conversion compared to the other catalysts. The molecular structures and summary table of the calculated and found molecular weights obtained from MALDI-ToF-MS analyses are provided in Fig. S30 and Table S1. Among these bases, only TEA showed no evidence of nucleophilic addition in the MALDI-ToF-MS analyses. The presence of the nucleophilic addition reactions can explain the discrepancy between the ^1^H-NMR and GPC results regarding acrylates conversion and monomer content. The base reacted monomers could exhibit similar retention times in GPC to those of unreacted monomers. MALDI-ToF-MS analyses reveal that these bases also react with higher molecular weight LCOs. The total concentration of these species is expected to be around 6 mol%, which should not exceed the initial concentration of the base catalyst in the reactions. In contrast, TEA follows a purely base-catalysed thiol-Michael addition pathway. Different from previous cases, the presence of only EDDET reacted MMs and DMs can also explain the disagreement between GPC and ^1^H-NMR analyses for TEA. However, our investigation highlights that TEA uniquely enables nucleophilic addition free thiol-Michael addition without side reactions, albeit at the cost of lower conversion.

Among these four catalysts, in order to avoid side products in the thiol-Michael addition reaction, results suggest that a tertiary amine such as TEA should be used. Besides the nucleophilic addition, the GPC and MALDI-ToF analyses of DBU reacted samples show a clear indication of a cleavage reaction. To prove the case, we have done additional experiments with non-reactive mesogenic (NRM) units sharing the same core molecular structure as DM (Fig. S31). We found that extra ^1^H-NMR signals were observed only in the case of the DBU catalysed reaction (Fig. S32). One of these signals corresponds to the presence of a carboxylic acid proton in the sample, indicating cleavage of the aromatic ester bond due to DBU.

We also experimented with a non-reactive derivative of a widely used chiral dopant and found that for DBU, DPA, and α-MBA extra peaks appeared in ^1^H-NMR analyses, which indicates cleavage when there are sequential aromatic ester bonds (Fig. S33–S38). These extra peaks were more significant in the presence of EDDET as the proton donor reactant, suggesting a cleavage reaction. Since TEA was promising as a highly selective thiol-Michael addition base catalyst, to improve the overall conversion, alternative aprotic solvents like acetone and DMF were investigated.^3^ However, no significant improvement was observed by using different aprotic solvents (Fig. S39). On the other hand, increasing the TEA concentration to 10.0 mol% and doubling the overall reactant concentration in DCM by halving the solvent volume led to a marked improvement in reaction yield. ^1^H-NMR analysis of the concentrated TEA catalysed reaction showed an acrylate conversion of 95 mol% in the thiol-Michael addition reaction after 24 h (Fig. S40). We did not observe extra peak formation or unusual peak shifts in ^1^H-NMR after increasing the overall concentration and hence we do not expect the aforementioned side reactions present in this batch. The GPC analysis of this concentrated reaction also supports the MALDI-ToF-MS findings that the TEA catalysed reaction batch also included some thiol-end oligomers, which are going to continue to grow with DMs or become end-capped with MMs.

**Fig. 4:**
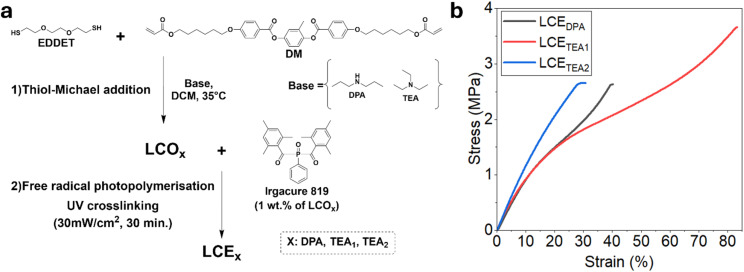
(a) Schematic of the model reaction to prepare LCEs. X represents the base used in thiol-Michael addition. (b) Stress–strain analyses of LCE_DPA_, LCE_TEA1_, and LCE_TEA2_.

Based on the above findings, we prepared LCEs (LCE_DPA_, LCE_TEA1_, and LCE_TEA2_) using 3 eq. of DM, 2 eq. of EDDET and DPA (6 mol%) or TEA (6 mol% or 10 mol% with doubled concentration) as the base (Fig. 4a). After the thiol Michael addition reaction, the solvent and free base were evaporated at room temperature, overnight. ^1^H NMR spectroscopy revealed the presence of acrylate endcapped oligomers with degrees of polymerization of 3.44, 2.20, and 3.05, for oligomers used for LCE_DPA_, LCE_TEA1_, and LCE_TEA2,_ respectively (Fig. S42–44). MALDI-ToF-MS analyses again revealed the formation of nucleophilic base adducts as side products but only when DPA was used as a base (Fig. S47). The degree of polymerization of LCE_TEA2_ agrees well with the feed ratio, assuming high conversion (*vide supra*). For LCE_TEA1_, a lower degree of polymerization is observed, possibly due to an incomplete thiol-Michael addition reaction, while LCE_DPA_ exhibits a higher degree of polymerization compared to LCE_TEA2_, which may be attributed to the formation of DPA-adduct products (*vide supra*). Subsequently, the oligomers were photopolymerized in the presence of a photoinitiator, to fabricate LCEs.^27^ Dynamic mechanical thermal analysis (DMTA) of LCE_DPA_, LCE_TEA1_, and LCE_TEA2_ showed different storage (*G*′) values of 48.9 MPa, 34.5 MPa, and 32.4 MPa, respectively (Fig. S48). The same trend was observed for the loss modulus (*G*″), with values of 18.6 MPa, 12.1 MPa, and 9.7 MPa (Fig. S49). The Young's modulus (*E*) values for the LCEs were determined to be 12 MPa, 10.0 MPa, and 9.4 MPa for LCE_TEA2_, LCE_TEA1_, and LCE_DPA_, respectively. The stress–strain data indicate that the LCEs become stiffer and mechanically stronger in the order LCE_DPA_ < LCE_TEA1_ < LCE_TEA2_. The crosslink density is most likely highest in LCE_TEA2_, as it contains a high concentration of acrylate groups prior to the crosslinking step. In contrast, LCE_DPA_ exhibits a lower crosslink density due to the formation of base-adduct products. In the case of LCE_TEA1_, the unreacted EDDET resulting from the incomplete thiol-Michael addition may act as a chain-transfer agent in the second photopolymerisation step, thereby reducing the effective crosslink density. These differences in crosslinking density are supported by the stress–strain behaviours of LCE_DPA_ and LCE_TEA1_, showing typical “flow” characteristic, whereas LCE_TEA2_ exhibits no flow behaviour (Fig. 4b). These data show that the catalyst (type and concentration) plays a decisive role in determining the mechanical properties of LCEs.

## Conclusions

This study demonstrates that apart from TEA, three commonly used bases for preparing LCOs and LCEs lead to the formation of nucleophilic base adducts as side products as identified with MALDI-ToF-MS analyses. Although thiol-Michael addition is regarded as a highly selective reaction, careful consideration of the choices is essential to achieve maximal selectivity. We attribute the absence of nucleophilic addition in the case of TEA to its tertiary amine structure. However, this advantage comes at the cost of a lower overall conversion in thiol-Michael addition. The conversion could be significantly improved to 95% by increasing the TEA catalyst concentration to 10 mol% of the reactants and the overall reactant concentration was set to approximately 0.5 M. The DMTA analyses of LCE_DPA_, LCE_TEA1_, and LCE_TEA2_ indicate that both the type and the concentration of the catalyst used in thiol-Michael addition directly influence the mechanical properties of the resulting LCEs.

Our results indicate that previously synthesized LCOs and LCEs prepared *via* base catalysed thiol-Michael addition may contain base adduct impurities. Consequently, the effective crosslink density may differ from the intended design as supported by the demonstrated LCEs. Overall, this work provides practical guidelines for the preparation of LCOs and LCEs, which might ultimately enhance the performance of the resulting stimuli responsive materials and devices by enabling greater control over the thiol-Michael addition reaction.

## Author contributions

R. Umut Dinc: writing – original draft; conceptualization (equal); investigation; visualization; and writing – review & editing (equal). Johan Lub: conceptualization (equal) and writing – review & editing (supporting). Xianwen Lou: investigation (supporting). Augustinus J. J. Kragt: supervision (supporting); conceptualization (equal); and writing – review & editing (supporting). Albert P. H. J. Schenning: funding acquisition (lead); resources (lead); conceptualization (equal); supervision (equal); and writing – review & editing (supporting).

## Conflicts of interest

There are no conflicts to declare.

## Supplementary Material

SC-OLF-D6SC03222B-s001

## Data Availability

Supplementary information (SI): characterization, equipment, and additional analyses. See DOI: https://doi.org/10.1039/d6sc03222b. The database of this publication is available free of charge at https://doi.org/10.5281/zenodo.19679221.
