# Supplementary material for: Unveiling the role of base catalysts in the thiol-Michael addition reaction in liquid crystal oligomers and liquid crystal elastomers
Source: Chem Sci. 2026 May 13;17(26):13007–12. doi: 10.1039/d6sc03222b (PMC13196339; doi:10.1039/d6sc03222b)
Supplement: SC-017-D6SC03222B-s001 [file SC-017-D6SC03222B-s001.pdf]

Supplementary Information (SI) for Chemical Science.
This journal is © The Royal Society of Chemistry 2026

Supporting Information

Unveiling the role of base catalysts in the thiol-Michael addition reaction in liquid crystal oligomers and liquid crystal elastomers

Materials

All commercial chemicals and solvents were used as received unless stated otherwise. Dichloromethane (DCM), ethanol (EtOH), isopropanol (IPA), acetone, tetrahydrofuran (THF), toluene were obtained from BioSolve (The Netherlands). 1,8-Diazabicyclo[5.4.0]undec-7-ene (DBU), triethylamine (TEA), dipropylamine (DPA), hydrochloric acid (HCl), potassium carbonate, magnesium sulphate, and chloroform-d_6 with tetramethyl silane (TMS) standard were obtained from Merck (Germany). 3,6-Dioxa-1,8-octanedithiol (EDDET), DL-1-Phenylethylamine (α-MBA) were obtained from TCI (Japan). 2-methyl-1,4-phenylene bis(4-((6-(acryloyloxy)hexyl)oxy)benzoate) (DM) was obtained from Daken Chemical (China). 4-(4-hexyloxybenzoyloxy)-3-methylphenyl 4-(3-acryloyloxypropyloxy)benzoate (MM) has been synthesized as stated in the literature before [1].

Characterisation

The thiol-Michael reactions were monitored by ^{1}H-NMR spectroscopy. ^{1}H-NMR spectra were recorded on a Bruker Avance Core iii 400 MHz and data analyses was performed using MestReNova software. The Matrix-assisted laser desorption/ionization time-of-flight mass spectrometry (MALDI-ToF-MS) measurements were performed on a Bruker Autoflex Speed MALDI-MS instrument using CHCA (α-cyano-4-hydroxycinnamic acid) and DCTB (trans-2-[3-(4-tert-butylphenyl)-2-methyl-2-propenylidene]malononitrile) as the matrices. MALDI-ToF-MS spectra were collected via instrument software and the raw data was plotted and analysed in Origin software. Gel Permeation Chromatography (GPC) was performed using a Shimadzu Prominence-i LC2030C 3D Liquid Chromatograph equipped with a photodiode array (PDA) detector and a refractive index (RI) detector. Two columns combined in series (A PLgel mixed-D column and a PLgel Mixed-C column, both are 300 × 7.5 mm i.d., 5 µm particles, Agilent Technologies, Amsterdam, the Netherlands), and THF at a flow rate of 1mL/min were used for the GPC measurements. The GPC data were collected via instrument software and the data analyses were done using Origin software. The differential scanning calorimetry (DSC) measurements were performed on a TA instruments DSC2500, in TA_{Zero} hermetic pans with a temperature rate of 10°C/min, between -50°C and 150°C for liquid crystal oligomers and -50°C and 200°C for liquid crystal elastomers under N_2 atmosphere. The data has been analysed in TRIOS software. Dynamic mechanical thermal analysis (DMTA) measurements were performed by using Q800 DMA from TA Instruments, operated in multifrequency-strain mode from 25°C to 150 °C with a rate of 5 °C/min, with applying a preload force of 0.0100 N. The oscillation amplitude and frequency were set to 15.0 µm and 1 Hz, respectively. The stress-strain measurements were done in stress-strain mode, with applying a preload force of 0.00100 N, operated at 25°C with a force ramp of 0.2 N/min set to maximum force of 18.0 N.

Synthesis of endcapped liquid crystal oligomers (LCOs)

155.83 mg (2 mol eq.) monoacrylate mesogen (MM), 93.50 mg (1 mol eq.) diacrylate mesogen RM82 (DM) were put in a 20 mL amber vial. In a separate vial, 50.67 mg (2 mol eq.) dithiol linker (EDDET) was dissolved in 2 mL dichloromethane (DCM), vortexed for 30 seconds and transferred into the amber vial via a glass pipette. The vial then washed out with additional 1

mL DCM and vortexed 30 seconds again. The contents was transferred into the amber vial too, achieving 3 mL solvent volume in total. The 20 mL amber vial was heated to 35°C by a hot plate while stirred at 300 RPM for 5 minutes. Then, the chosen catalyst was added (0.3 mol eq.) to the vial at t_0 = 0 h. After addition of the catalyst, the amber vial was gently purged with argon for 30 s and then mechanically closed with a PTFE lined screw cap. The reactions were monitored by ^{1}H-NMR spectroscopy with samples taken up to 48 hours after addition of the catalyst. In remove the free base catalyst prior to MALDI-ToF-MS, the reaction mixtures were treated with 2M HCl and saturated potassium carbonate (K_2CO_3) after 48 h. Each work up step was performed twice. After extraction with brine, the organic layers were dried over magnesium sulphate ($MgSO_4$), followed by drying in a vacuum oven at 50°C overnight.

Free acrylate conversion calculation in the thiol-Michael addition

The free acrylate conversion at each time point has been calculated with respect to the following ^{1}H-NMR peaks. The free acrylate peaks at 6.42 ppm, 6.13 ppm and 5.83 ppm at any time point were integrated and the sum of these integrals divided by the integrals at t_0 (feed). The ^{1}H-NMR peaks were normalized with respect to 8.15 ppm peak which corresponds to the two benzene ring protons which are not contributing to thiol-Michael addition. The conversion then calculated with respect to the equation below:

$$Free - acrylate\ conversion\ (\%) = \left(1 - \frac{(\int 6.42\, ppm + 6.13\, ppm + 5.83\, ppm)_t}{(\int 6.42\, ppm + 6.13\, ppm + 5.83\, ppm)_{t_0}}\right) * 100$$

Where "t" is the time stamp when the ^{1}H-NMR sample was collected. For example, in Figure S1, the sum of the integrals of the remaining free acrylates peaks are 0.38 (0.12 + 0.13 + 0.13) while the integrals are normalized with respect to 8.15 ppm peak assuming 3 mesogenic units in the feed (each mesogenic unit has 4 protons for this peak). When the feed is completely unreacted, the sum of the free acrylate integrals should be 12 when they are normalized in the same way. Therefore, the free acrylate conversion should be around 96.8%.

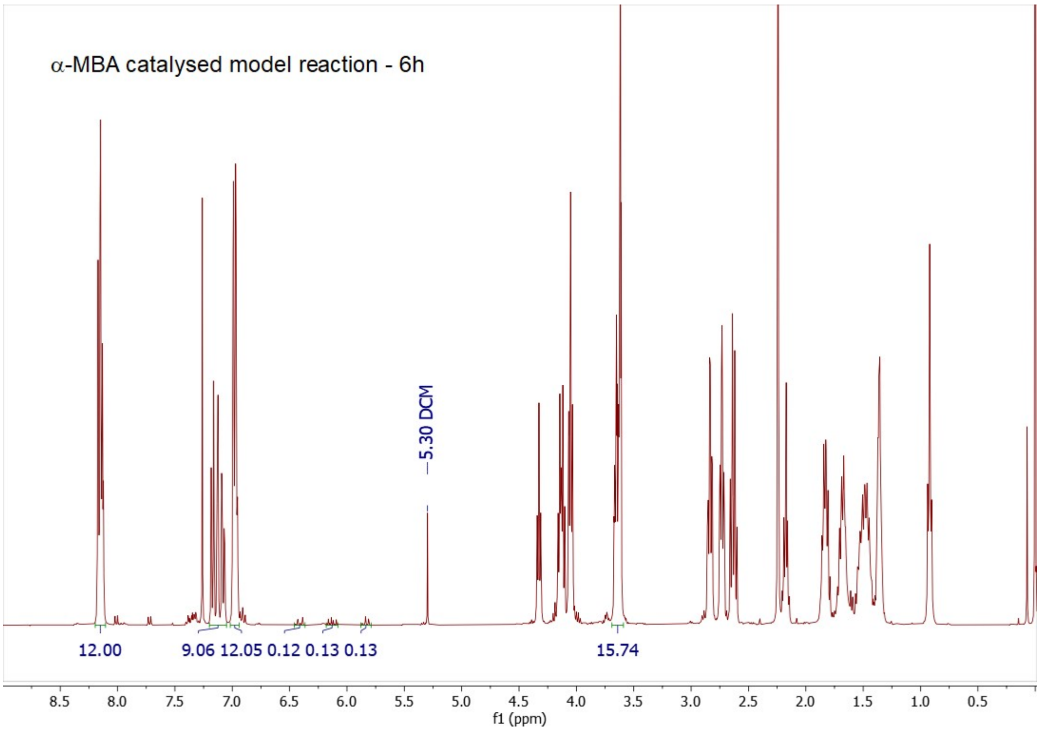

Figure S1 ^{1}H-NMR spectrum of the α-MBA catalysed reaction after 6 hours.

DPA catalysed thiol-Michael addition

^{1}H-NMR Spectra

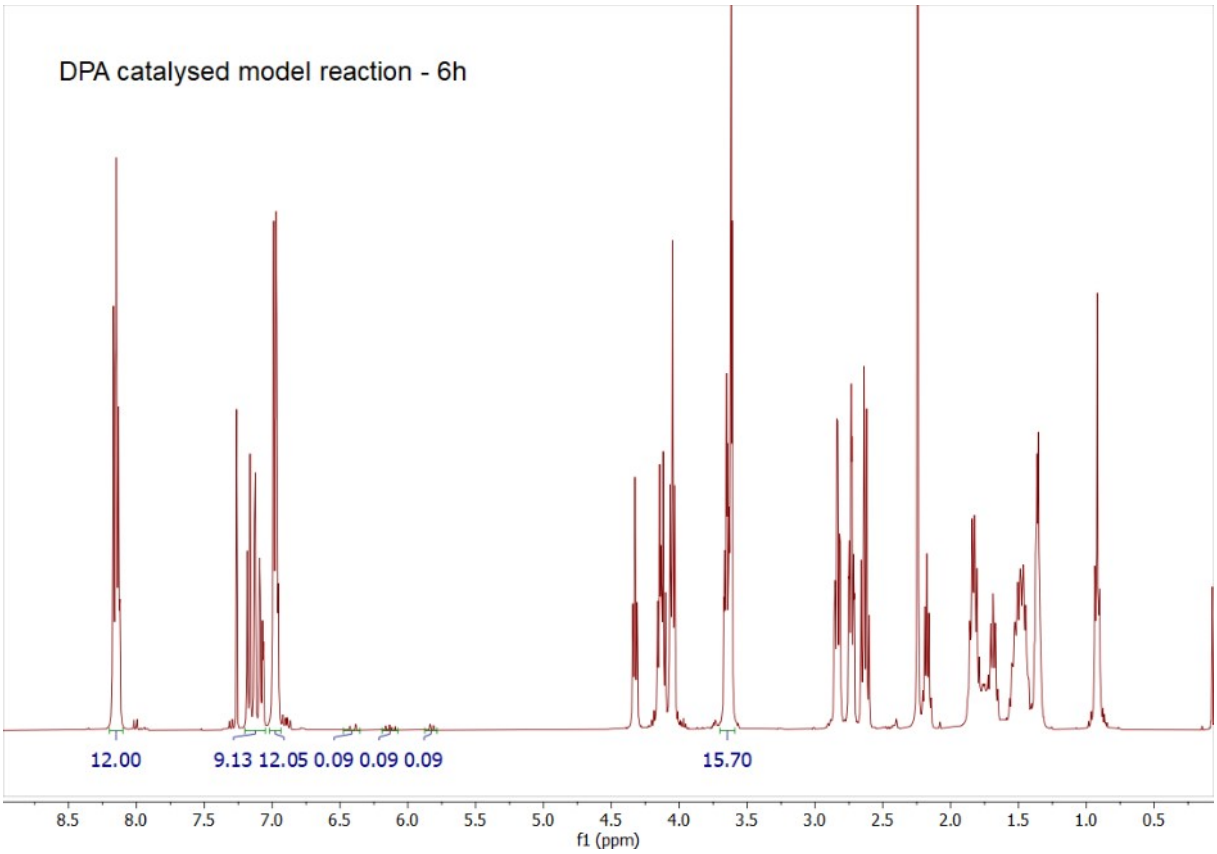

Figure S2. ^{1}H-NMR spectrum of DPA catalysed reaction after 6 hours.

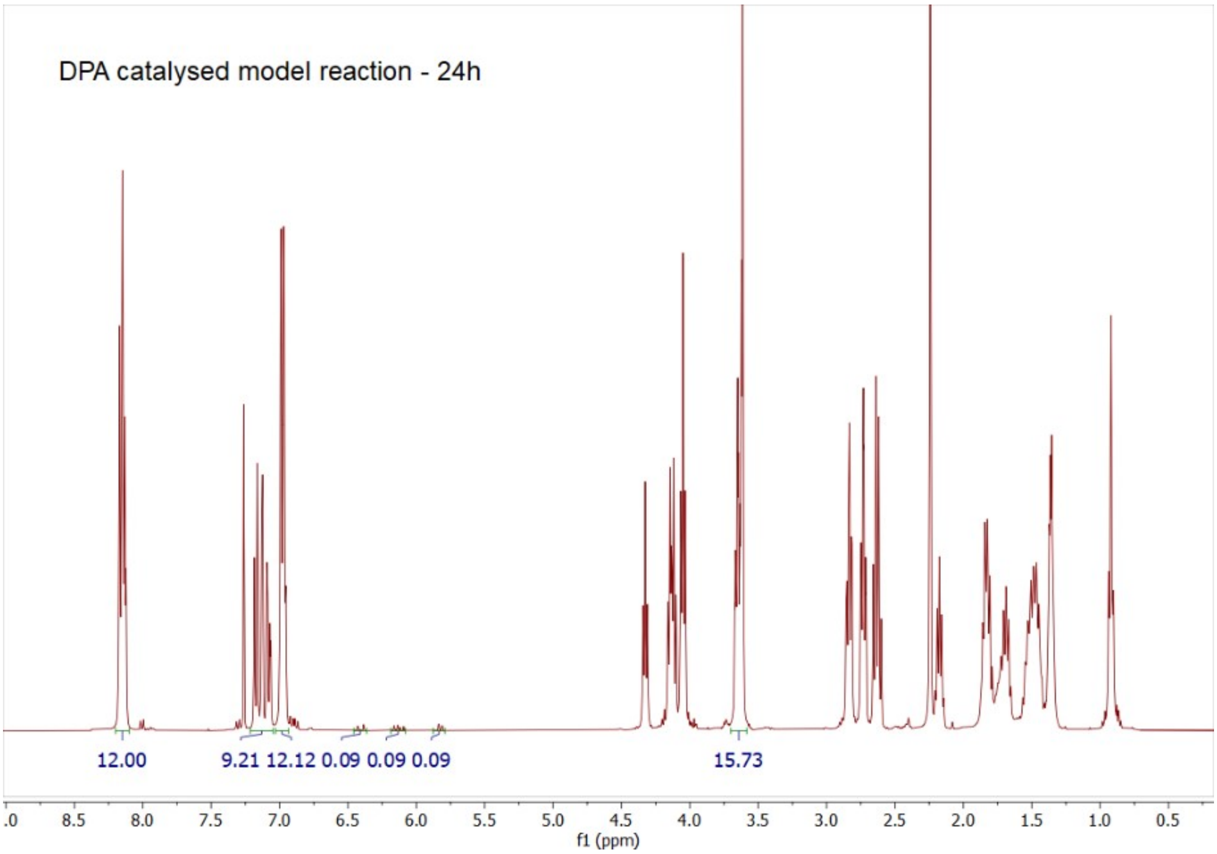

Figure S3. ^{1}H-NMR spectrum of DPA catalysed reaction after 24 hours.

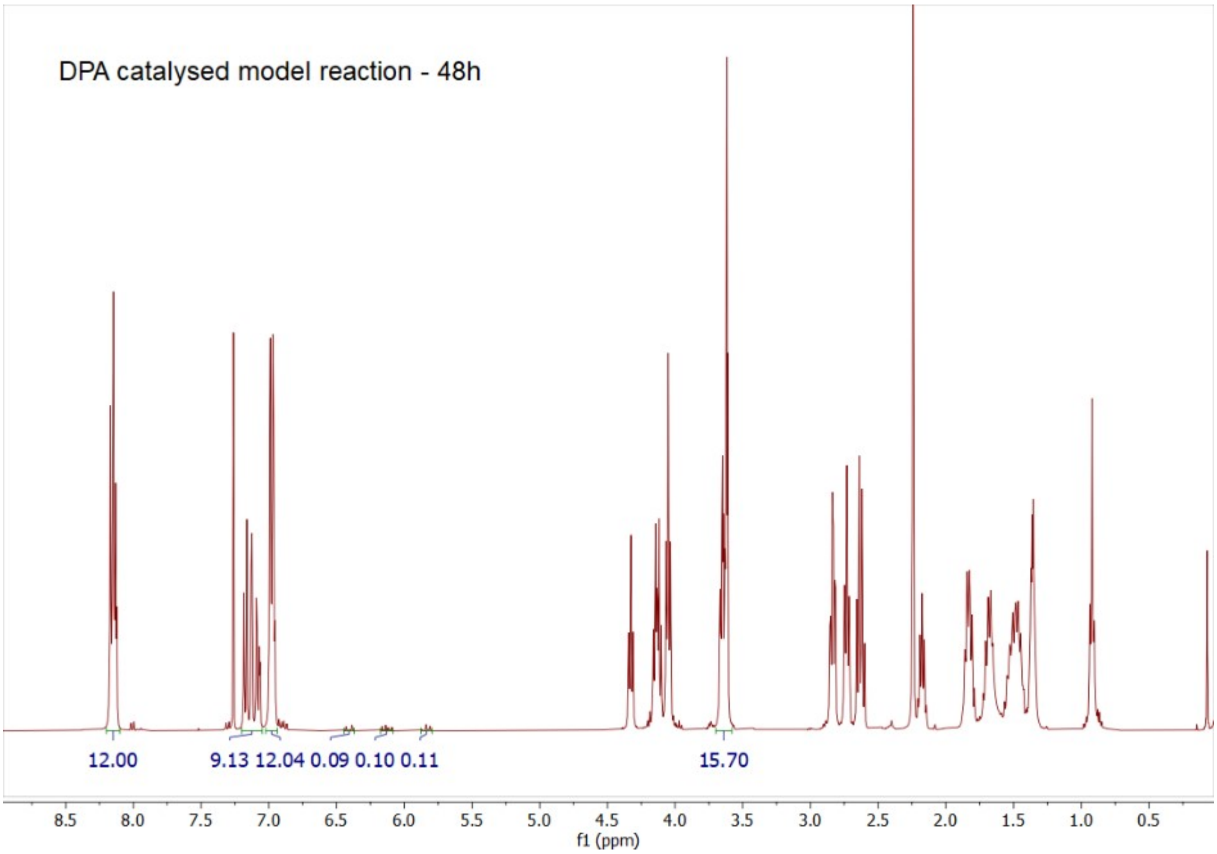

Figure S4. ^{1}H-NMR spectrum of DPA catalysed reaction after 48 hours.

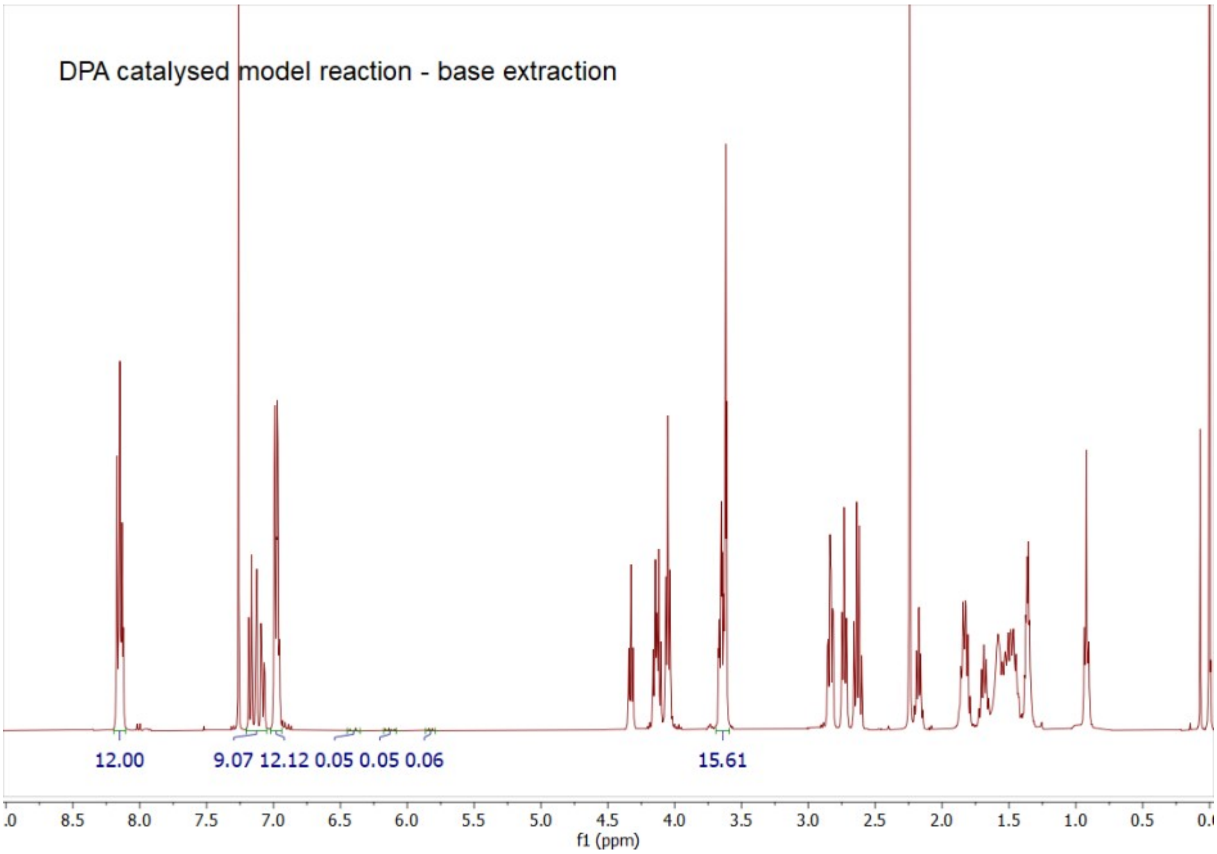

Figure S5. ^{1}H-NMR spectrum of DPA catalysed reaction after 48 hours and base extraction.

GPC profile after 48 hours

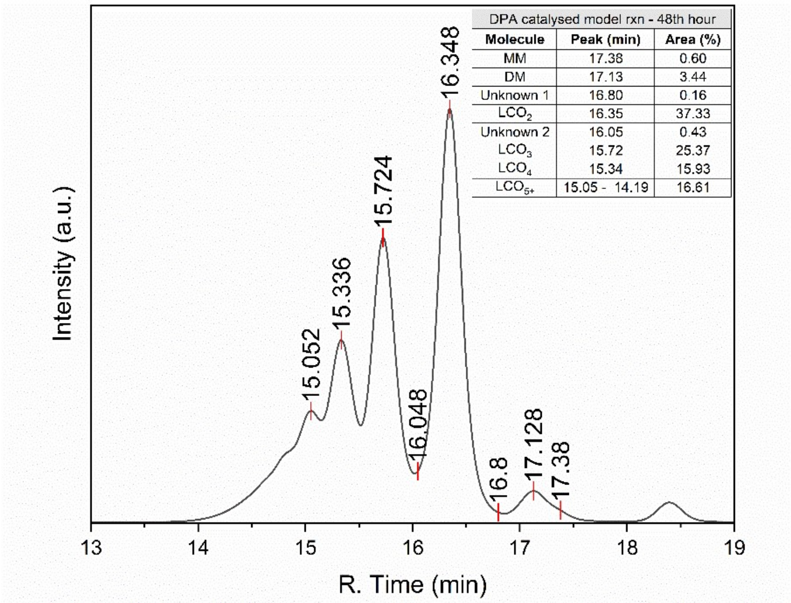

Molecule	Peak (min)	Area (%)
MM	17.38	0.60
DM	17.13	3.44
Unknown 1	16.80	0.16
LCO_2	16.35	37.33
Unknown 2	16.05	0.43
LCO_3	15.72	25.37
LCO_4	15.34	15.93
LCO_{5+}	15.05 - 14.19	16.61

Figure S6. GPC profile of the DPA catalysed reaction after 48 h.

GPC profiles as function of reaction time

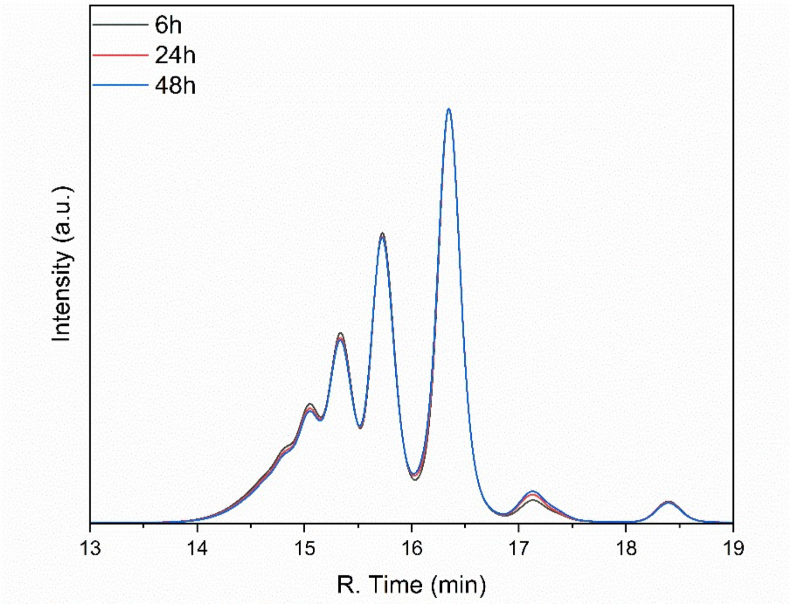

Figure S7. GPC profiles of DPA catalysed reaction at different time marks, showing an increase in 17.2 min (DM) signal while deacrease in higher M_w LCO signals with respect to time.

MALDI-ToF-MS spectrum

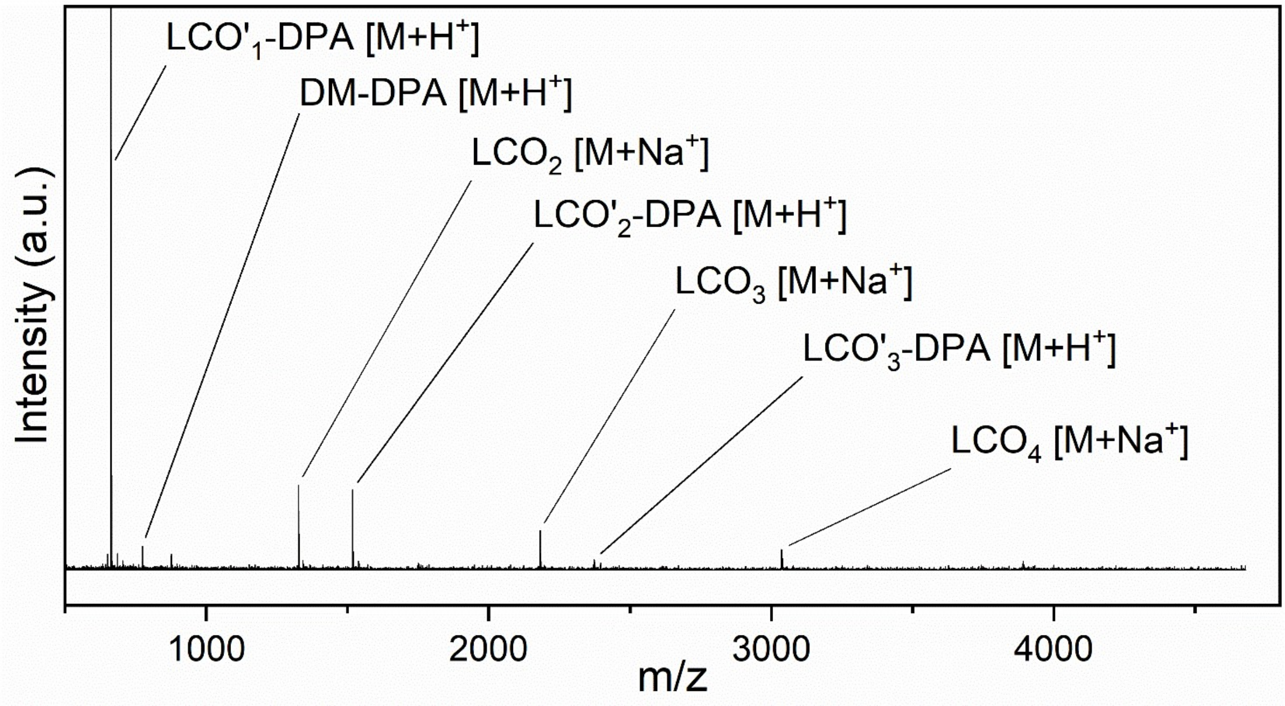

Figure S8 MALDI-ToF-MS spectrum of DPA catalysed sample.

α-MBA catalysed thiol-Michael addition

^{1}H-NMR Spectra

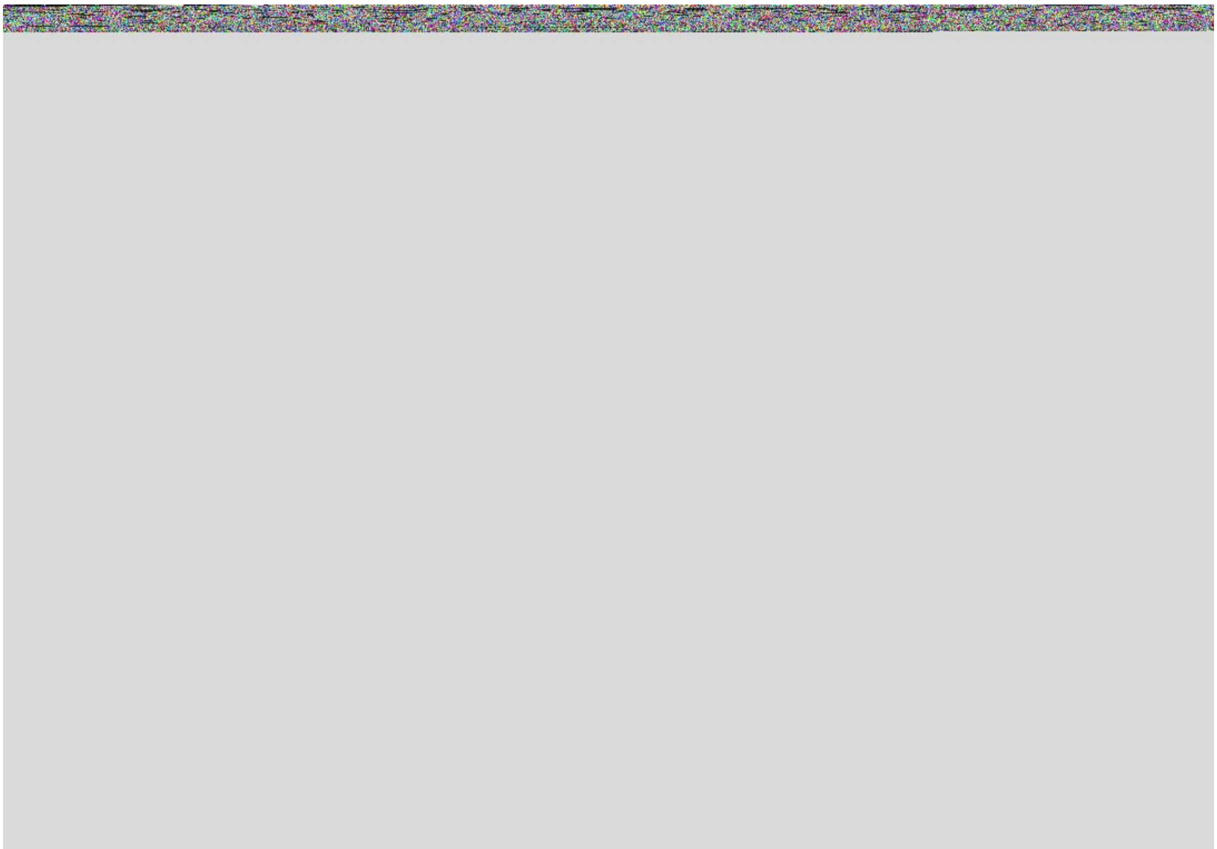

Figure S9 ^{1}H-NMR spectrum of α-MBA catalysed reaction after 6 hours.

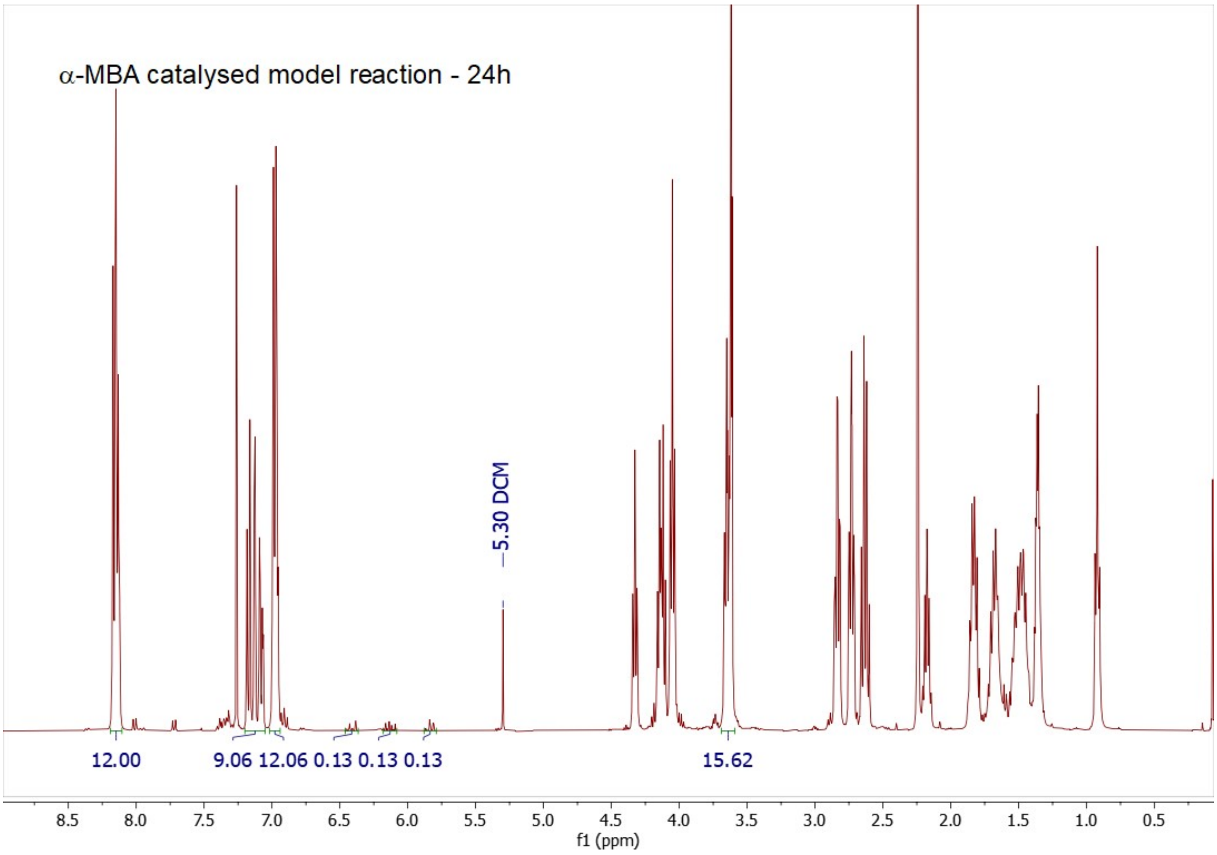

Figure S10 ^{1}H-NMR spectrum of α-MBA catalysed reaction after 24 hours.

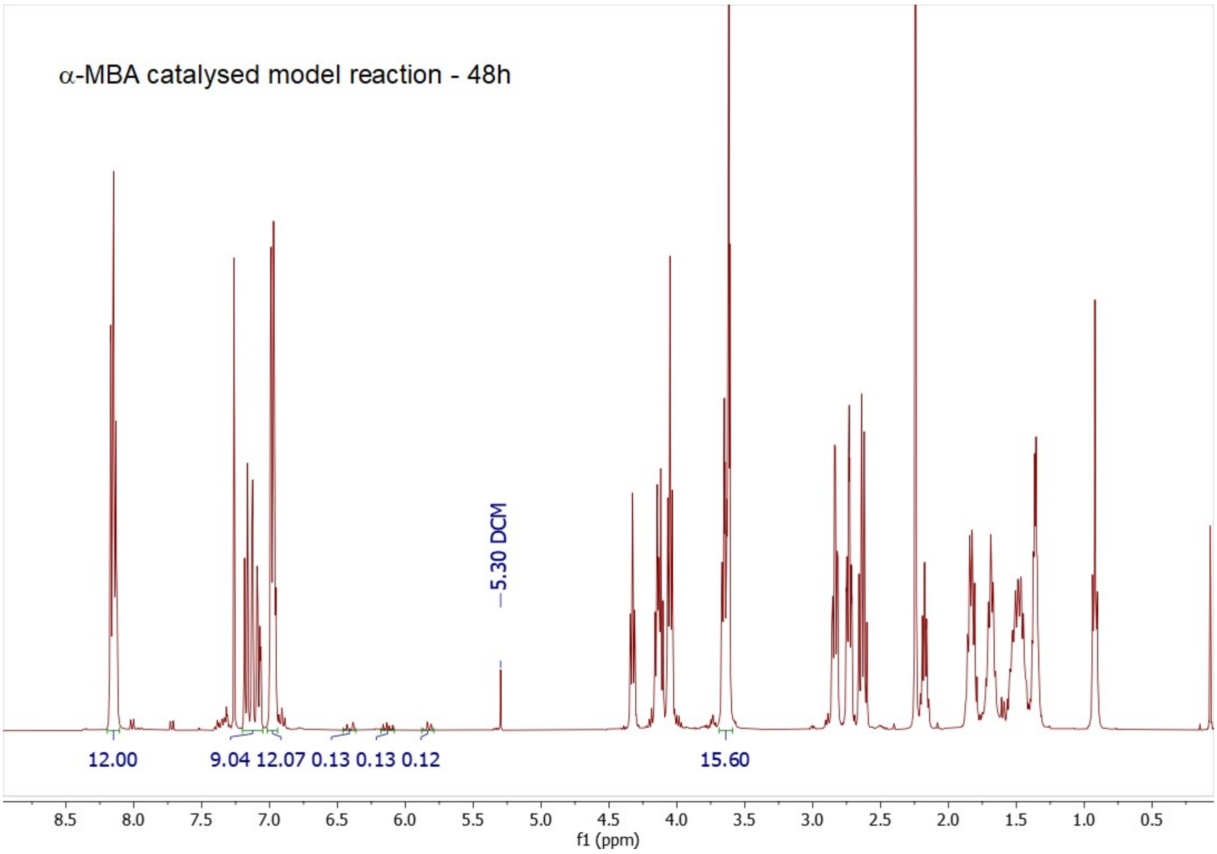

Figure S11 ^{1}H-NMR spectrum of α-MBA catalysed reaction after 48 hours.

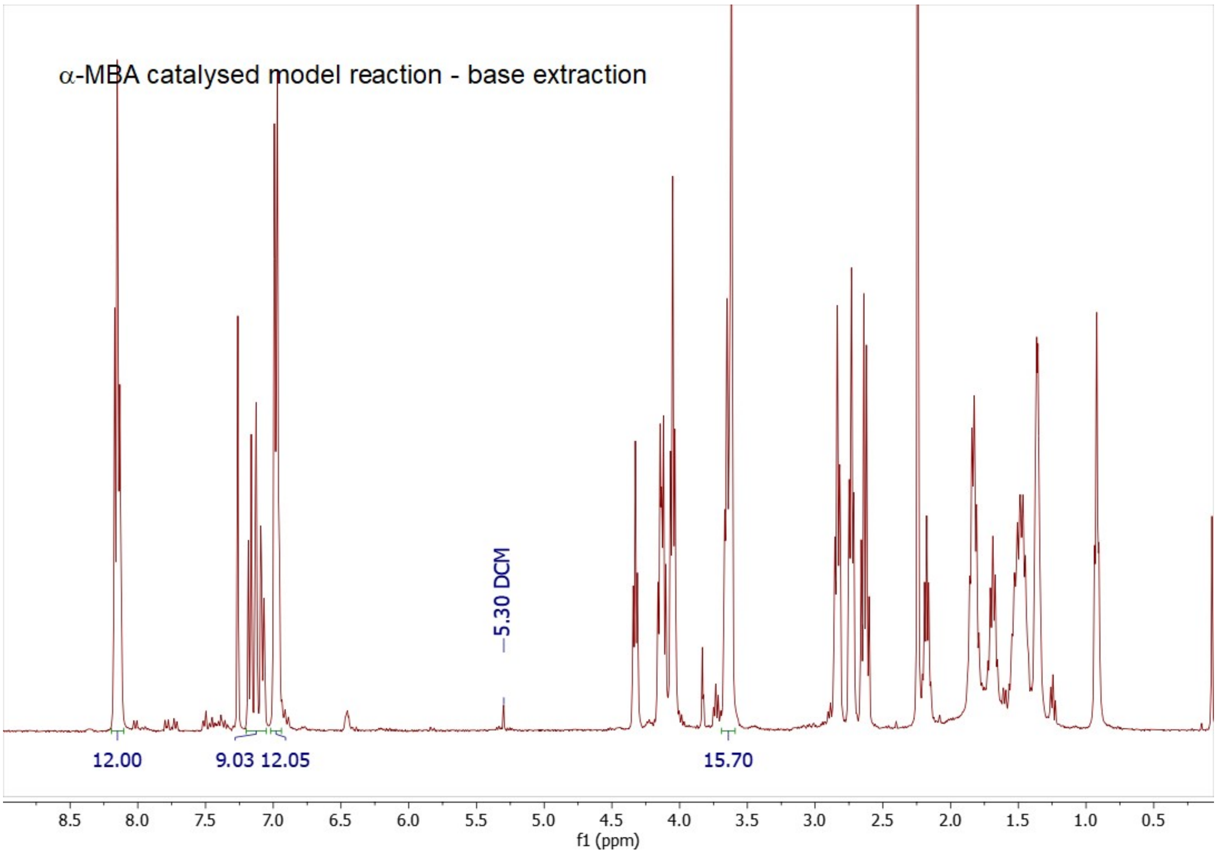

Figure S12 ^{1}H-NMR spectrum of α-MBA catalysed reaction after 48 hours and base extraction.

GPC profile after 48 hours

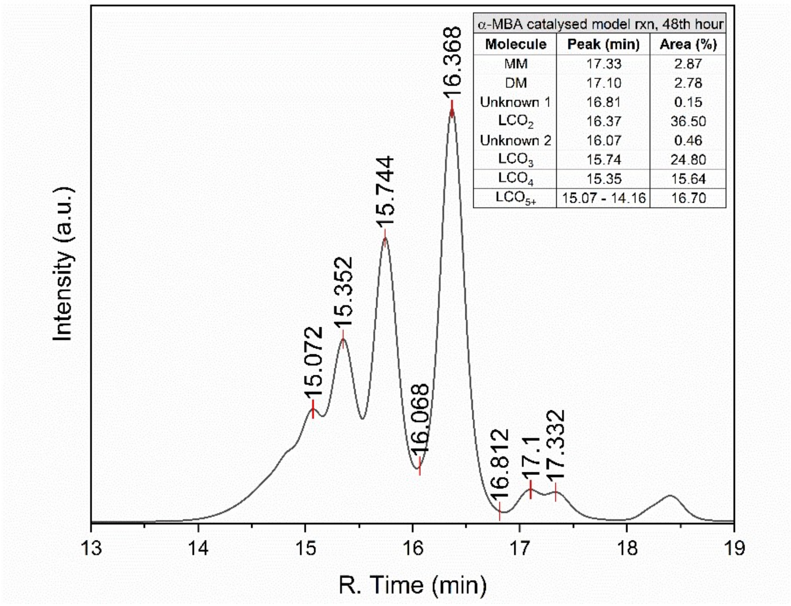

Figure S13 GPC profile of α-MBA catalysed reaction after 48th hour mark.

GPC profiles as function of reaction time

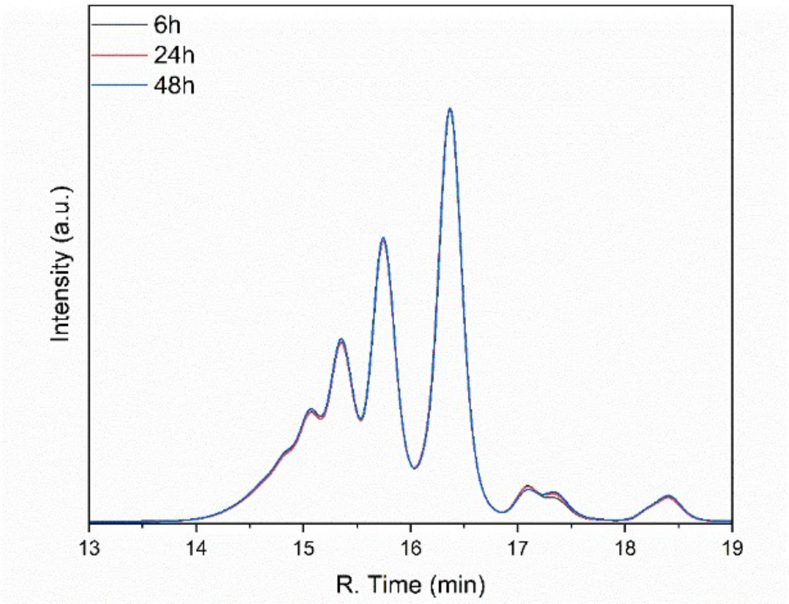

Figure S14 GPC profiles of α-MBA catalysed reaction at different time marks showing no significant change after 6th hour mark.

MALDI-ToF-MS spectrum

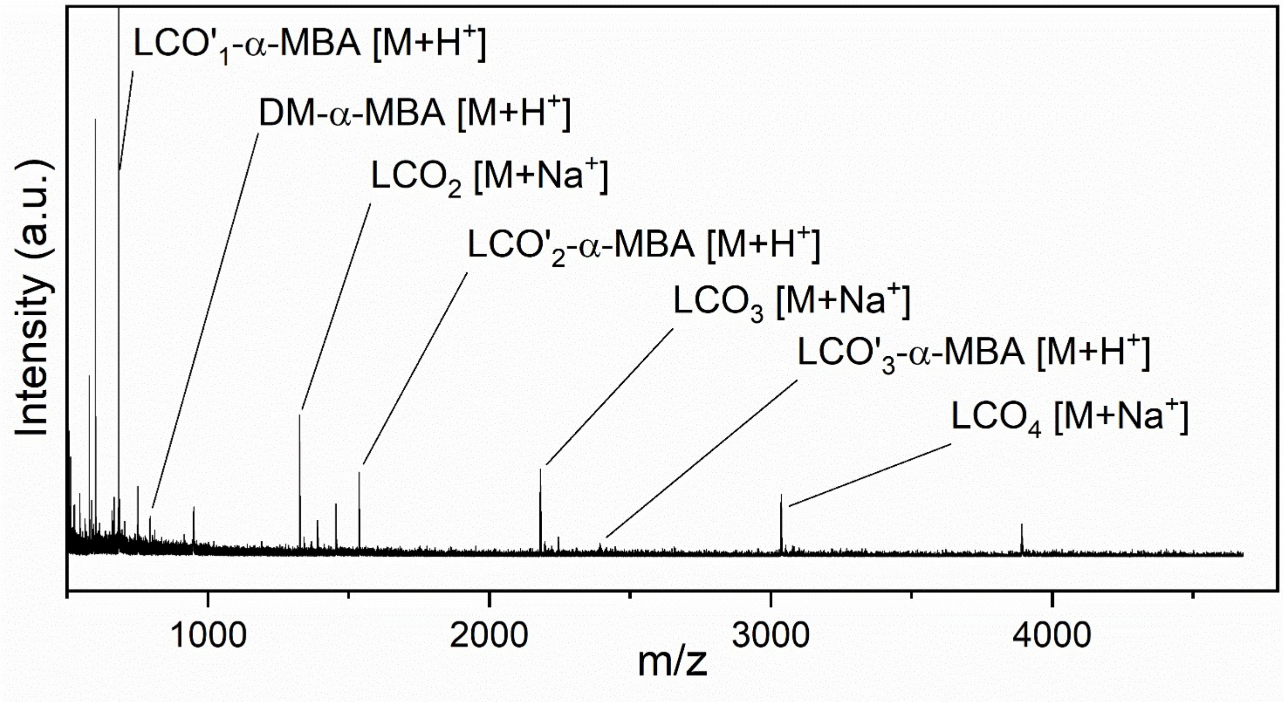

Figure S15 MALDI-ToF-MS spectrum of α-MBA catalysed sample.

DBU catalysed thiol-Michael addition

^{1}H-NMR Spectra

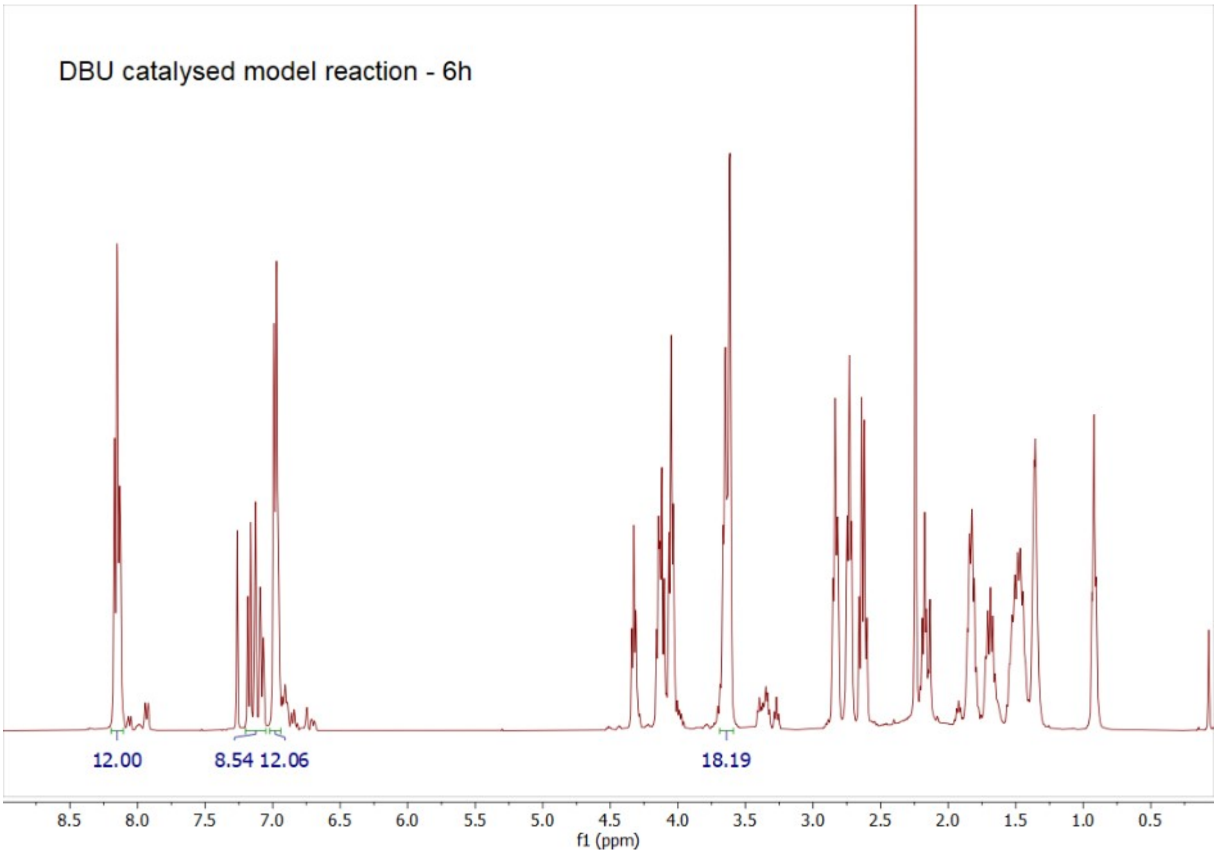

Figure S16 ^{1}H-NMR spectrum of DBU catalysed reaction after 6 hours.

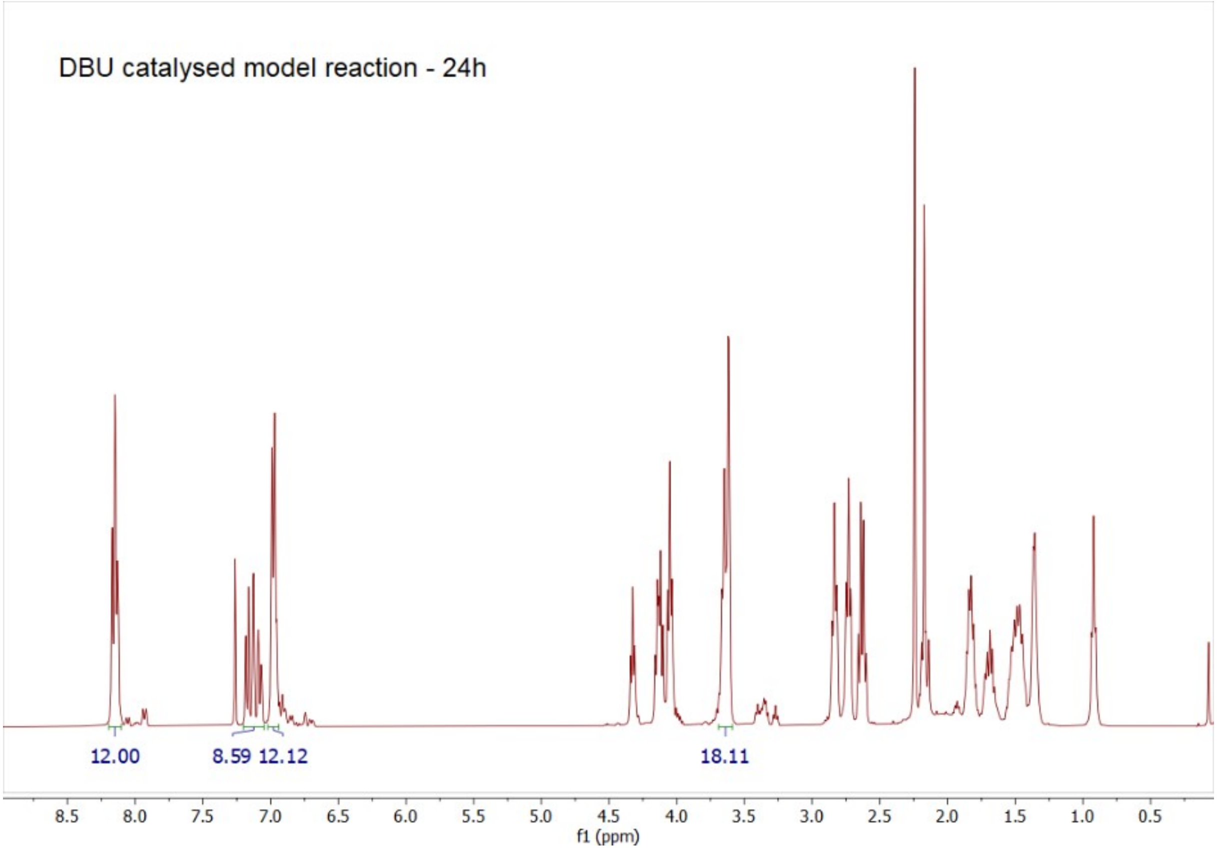

Figure S17 1H-NMR spectrum of DBU catalysed reaction after 24 hours.

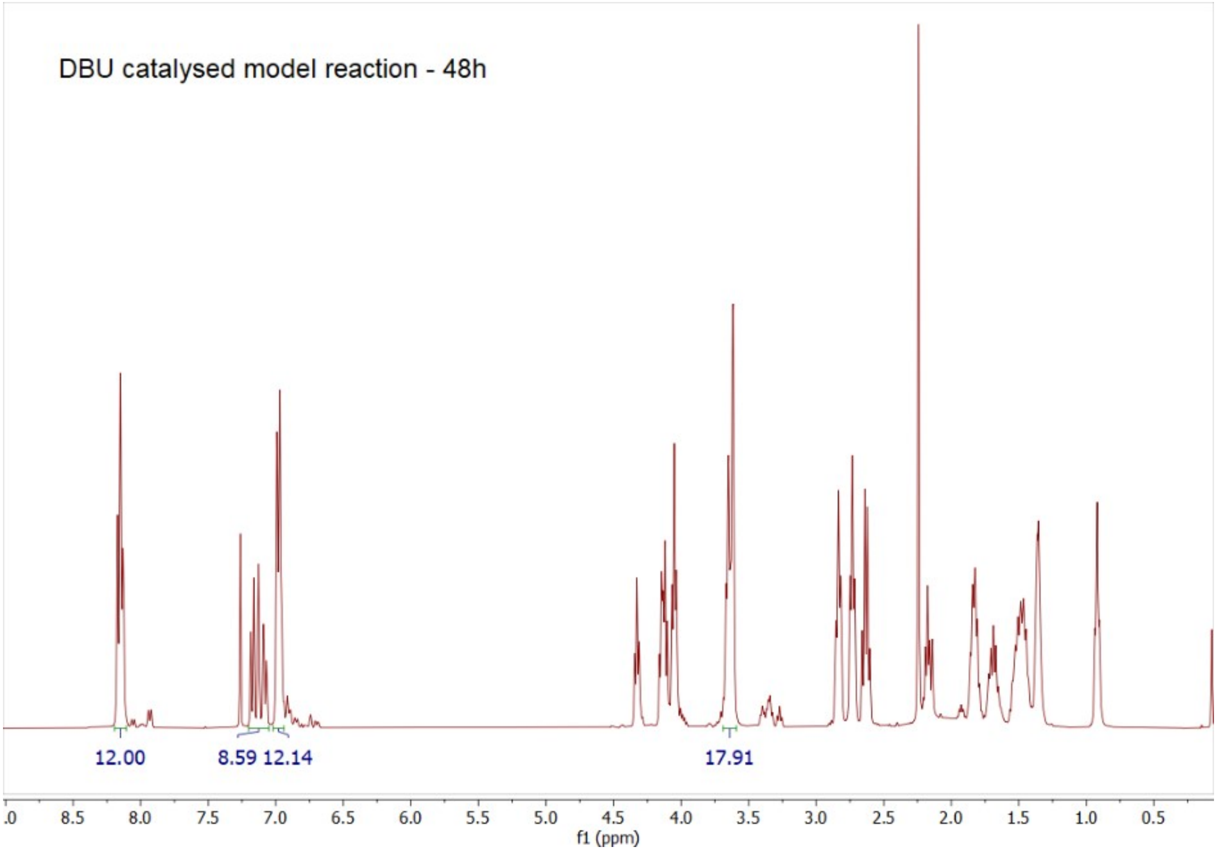

Figure S18 1H-NMR spectrum of DBU catalysed reaction after 48 hours.

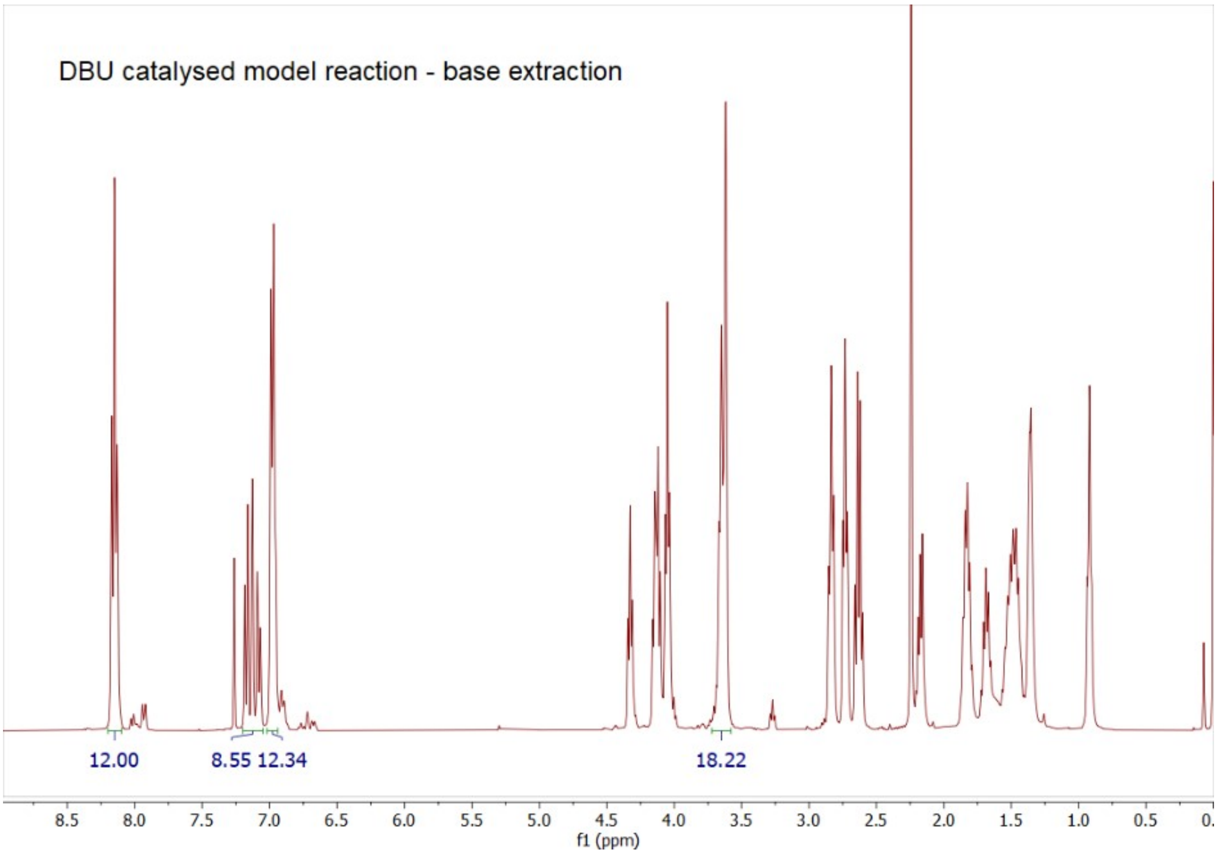

Figure S19 ^{1}H-NMR spectrum of α-MBA catalysed reaction after 48 hours and base extraction.

GPC profile after 48 hours

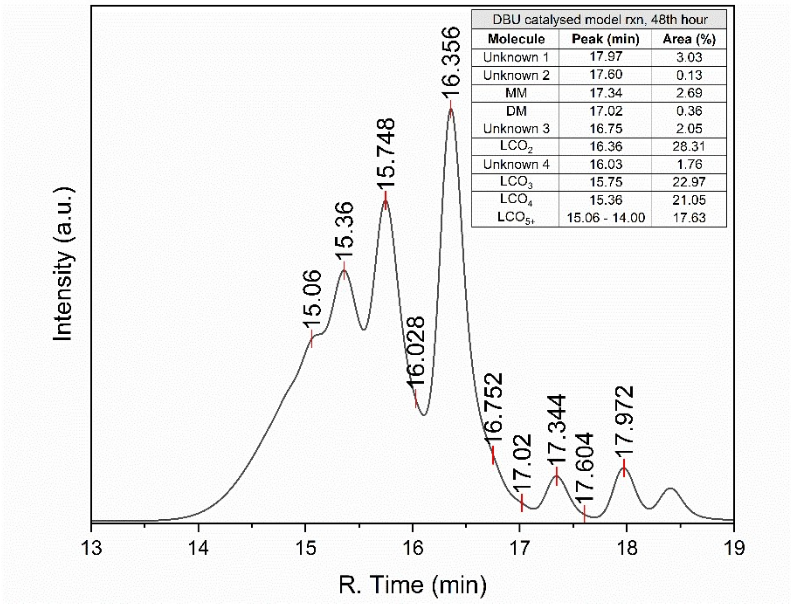

DBU catalysed model rxn, 48th hour		
**Molecule**	**Peak (min)**	**Area (%)**
Unknown 1	17.97	3.03
Unknown 2	17.60	0.13
MM	17.34	2.69
DM	17.02	0.36
Unknown 3	16.75	2.05
LCO_2	16.36	28.31
Unknown 4	16.03	1.76
LCO_3	15.75	22.97
LCO_4	15.36	21.05
LCO_{5+}	15.06 - 14.00	17.63

Figure S20 GPC profile of DBU catalysed reaction after 48th hour mark.

GPC profiles as function of reaction time

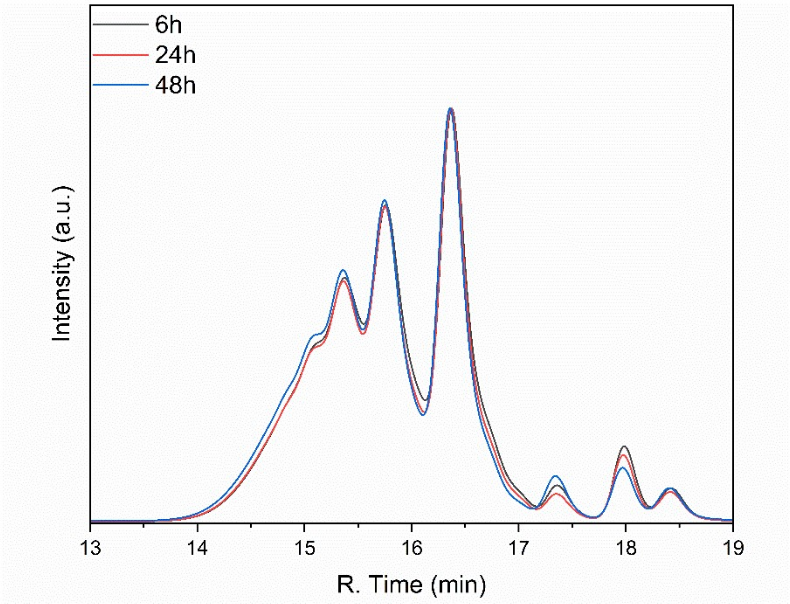

Figure S21 GPC profiles of DBU catalysed reaction at different time marks, showing a change in 18.0 min, 17.2 min (DM) and higher M_w LCO signals with respect to time.

MALDI-ToF-MS spectrum

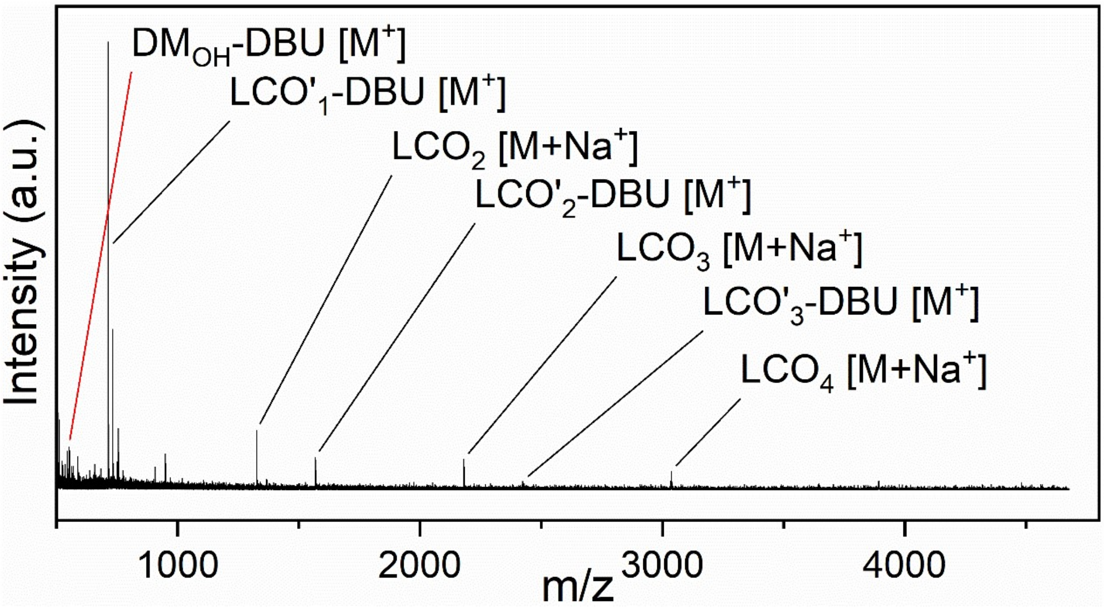

Figure S22 MALDI-ToF-MS spectrum of DBU catalysed sample.

TEA catalysed thiol-Michael addition

1H-NMR Spectra

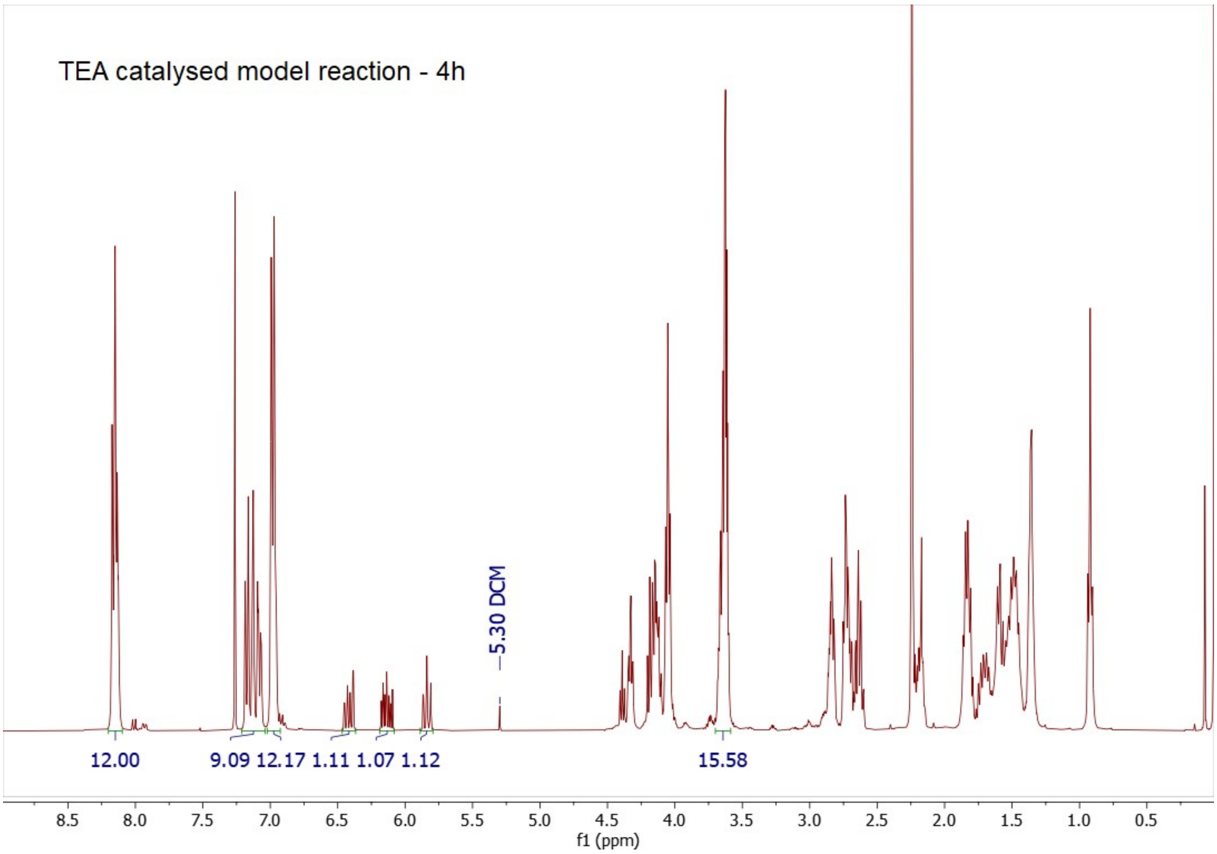

Figure S23 ^{1}H-NMR spectrum of TEA catalysed reaction after 4 hours.

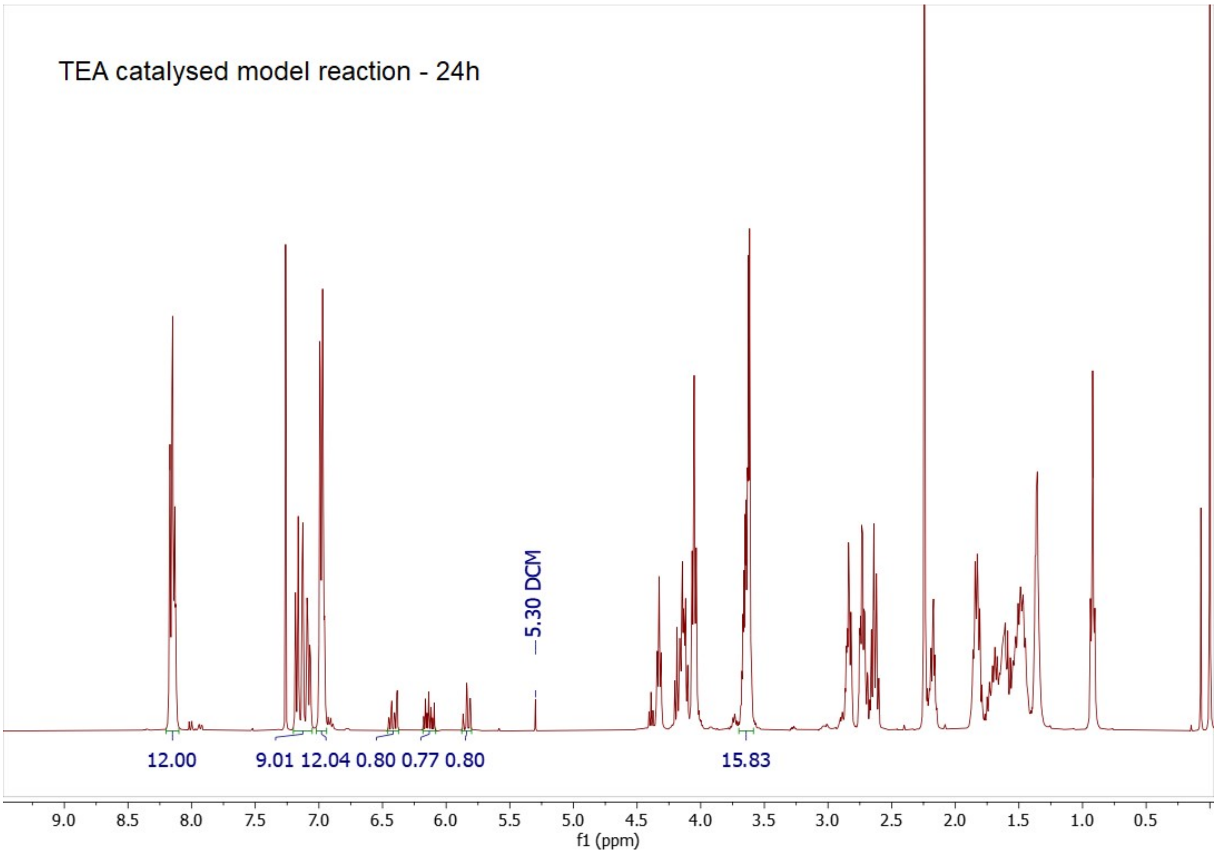

Figure S24 ^{1}H-NMR spectrum of TEA catalysed reaction after 24 hours.

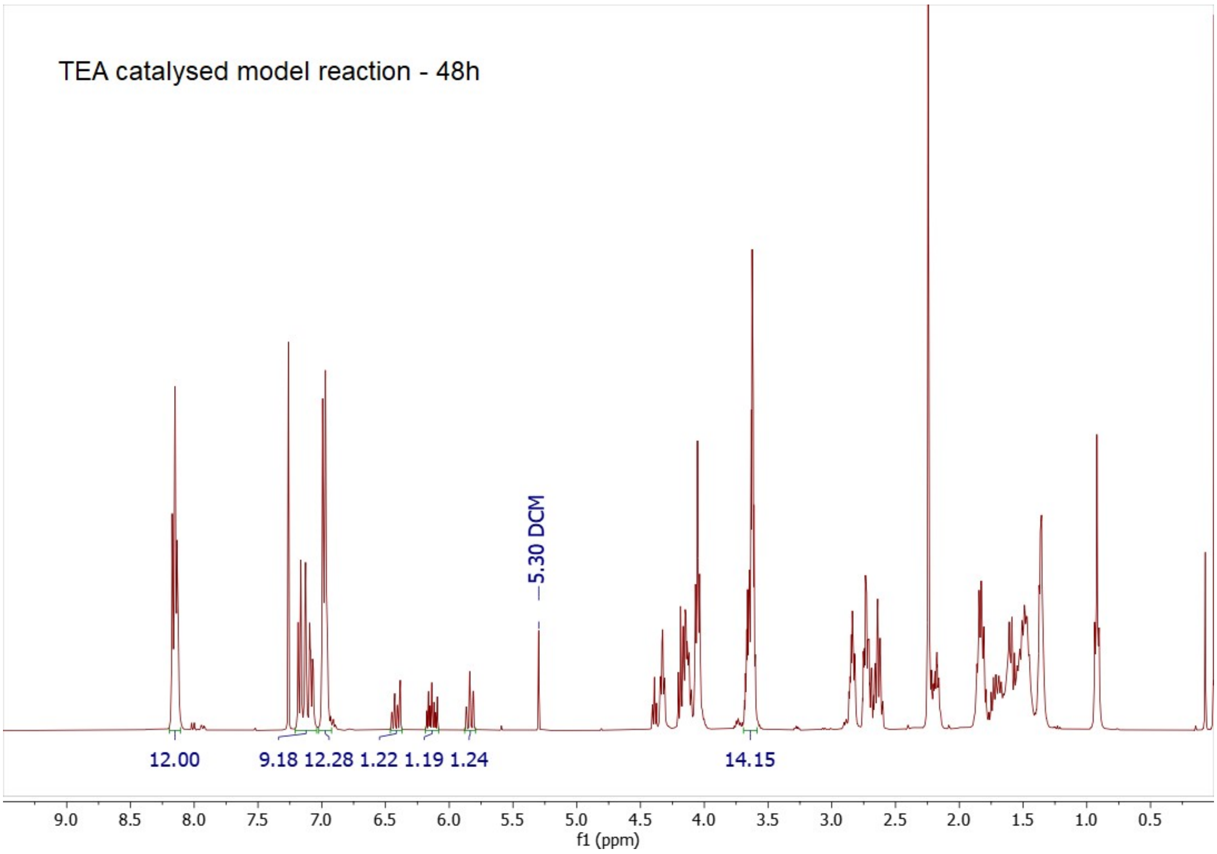

Figure S25 ^{1}H-NMR spectrum of TEA catalysed reaction after 48 hours.

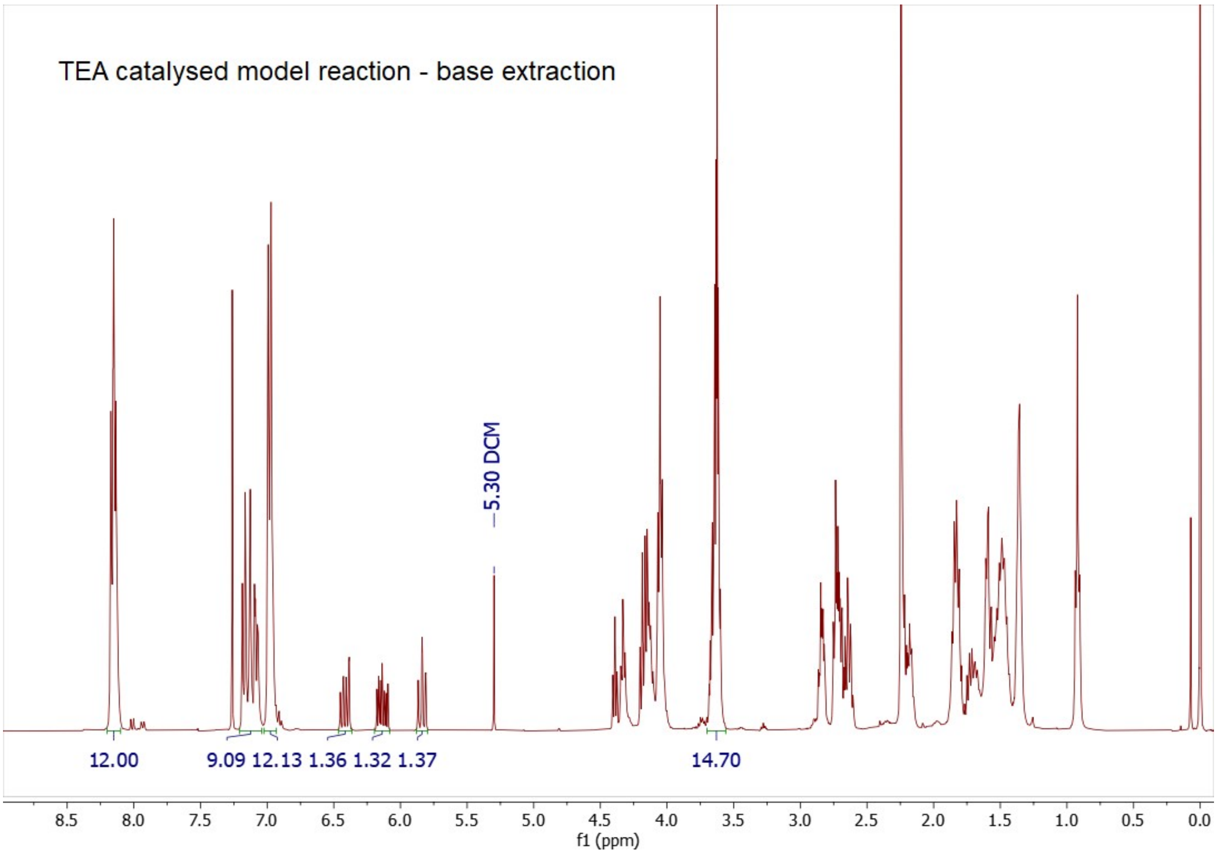

Figure S26 ^{1}H-NMR spectrum of TEA catalysed reaction after 48 hours and base extraction.

GPC profile after 48 hours

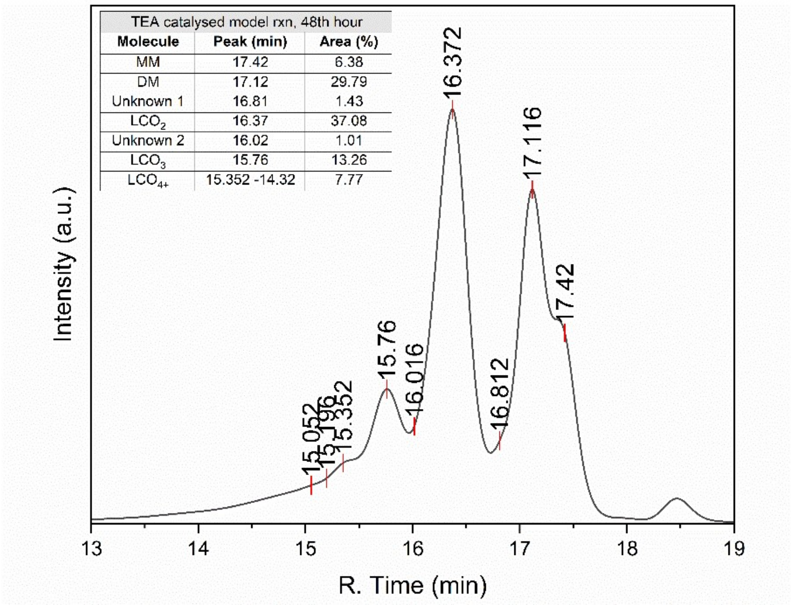

Figure S27 GPC profile of TEA catalysed reaction after 48th hour mark.

GPC profiles as function of reaction time

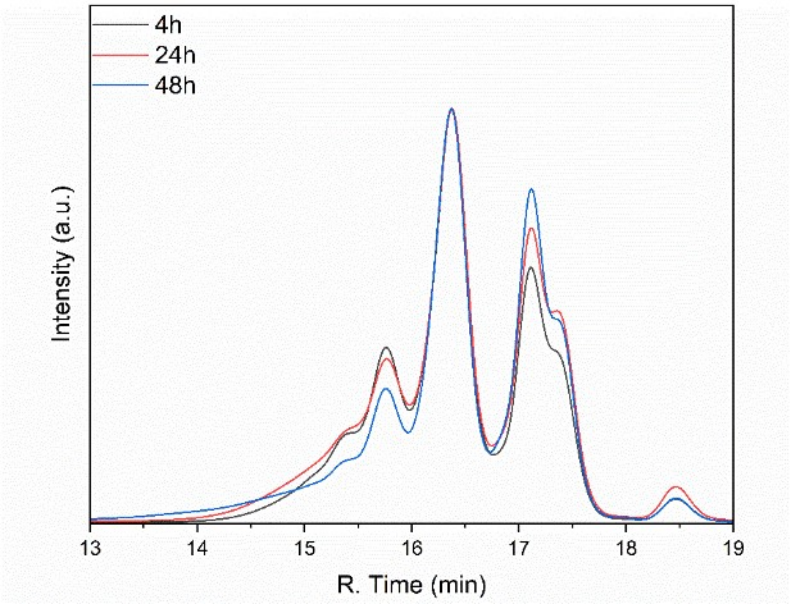

Figure S28 GPC profiles of TEA catalysed reaction at different time marks, showing an increase for 17.2 (DM) and 17.4 min (MM) signals whereas decrease for higher M_w LCO signals with respect to time.

MALDI-ToF-MS spectrum

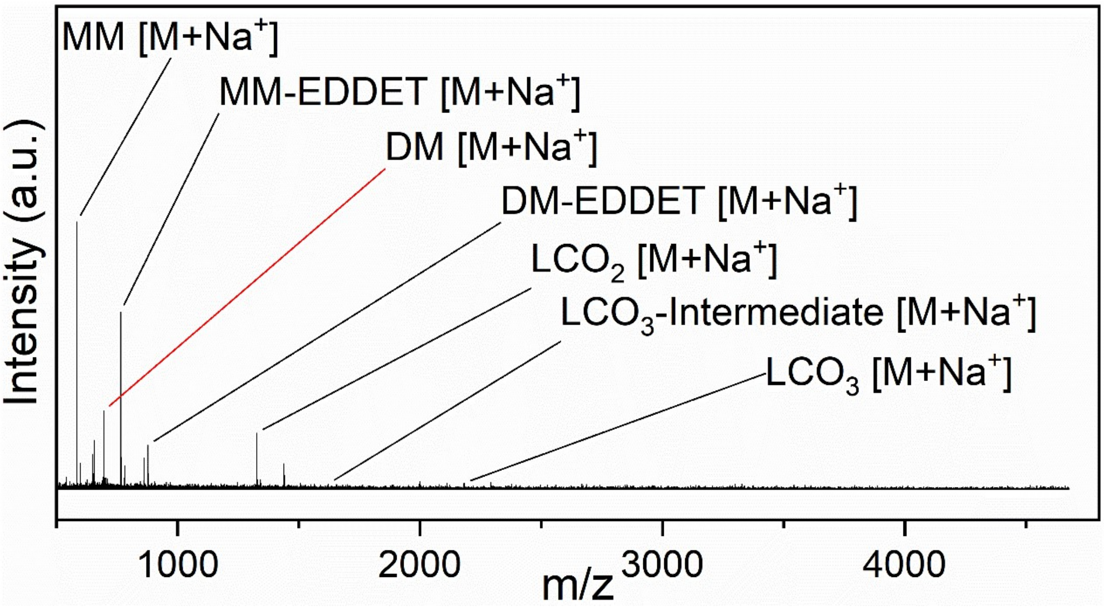

Figure S29 MALDI-ToF-MS spectrum of TEA catalysed sample.

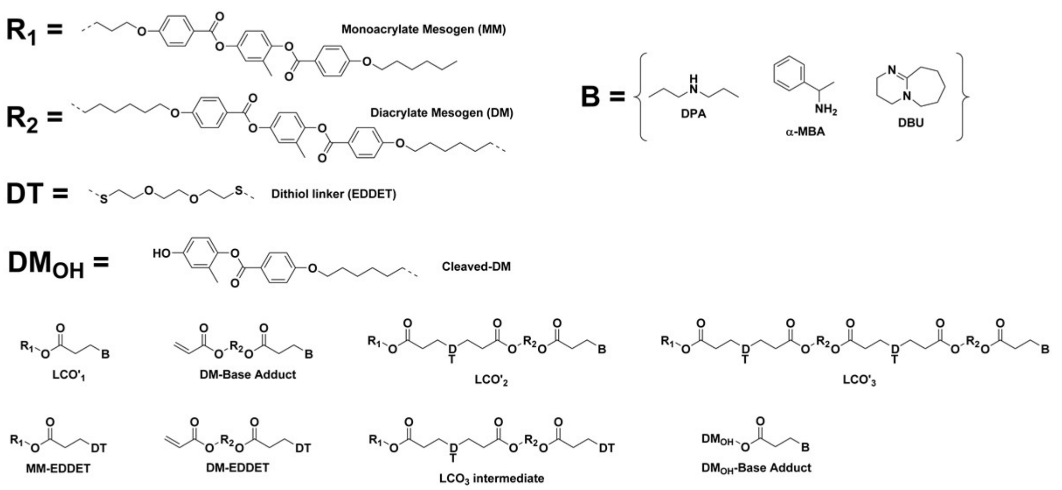

Figure S30 Molecule structures of the base adducts.

Table S1 MALDI-ToF-MS signals found in the samples in CHCA or DCTB matrices. *with Na adduct. **with H adduct

	[M + Na$^+$]* or [M + H$^+$]** or [M$^+$]	DPA	α-MBA	DBU	TEA
**MM**	Calculated	583.23*			
	Found	-	-	-	583.22*
**DM**	Calculated	695.28*			
	Found	-	-	-	695.28*
**MM-EDDET**	Calculated	-	-	-	765.27*
	Found	-	-	-	765.26*
**DM-EDDET**	Calculated	-	-	-	877.33*
	Found	-	-	-	877.31*
**LCO'$_1$**	Calculated	662.37**	682.34**	713.38	-
	Found	662.35**	682.34**	713.37	-
**DM-Base Adduct**	Calculated	774.42**	794.39**	-	-
	Found	774.40**	794.37**	-	-
**LCO$_2$**	Calculated	1325.52*			
	Found	1325.49*	1325.49*	1325.49*	1325.50*
**LCO$_3$**	Calculated	2180.86*			
	Found	2180.76*	2180.80*	2180.78*	2180.86*
**LCO$_3$-intermediate**	Calculated	-	-	-	1619.61*
	Found	-	-	-	1619.56*
**LCO'$_2$**	Calculated	1516.71**	1536.67**	1567.72	-
	Found	1516.65**	1536.68**	1567.68	-
**LCO$_4$**	Calculated	3035.19*			
	Found	3035.06*	3035.06*	3035.09*	-
**LCO'$_3$**	Calculated	2372.05*	2392.02*	2423.06	-
	Found	2372.03*	2392.01*	2423.00	-
**DM$_{OH}$-Base Adduct**	Calculated	-	-	551.31	-
	Found	-	-	551.29	-

Cleaving reactions between base catalysts and non-reactive mesogen (NRM) and non-reactive chiral dopant (NRCD)

The cleaving reaction was further investigated by testing the stability of a similar liquid crystals molecule and a widely used chiral dopant, excluding the reactive acrylate end groups as shown in **Figure S31**. We monitored the stability of these molecules by ^{1}H-NMR analyses, in the presence of the bases, with and without the dithiol (EDDET), up to 48 hours. The total concentration of the feed was kept around 150 mM. The mol equivalence of the NRM, EDDET and base was kept at 1. The NRM has been synthesized as stated in the literature before[2].

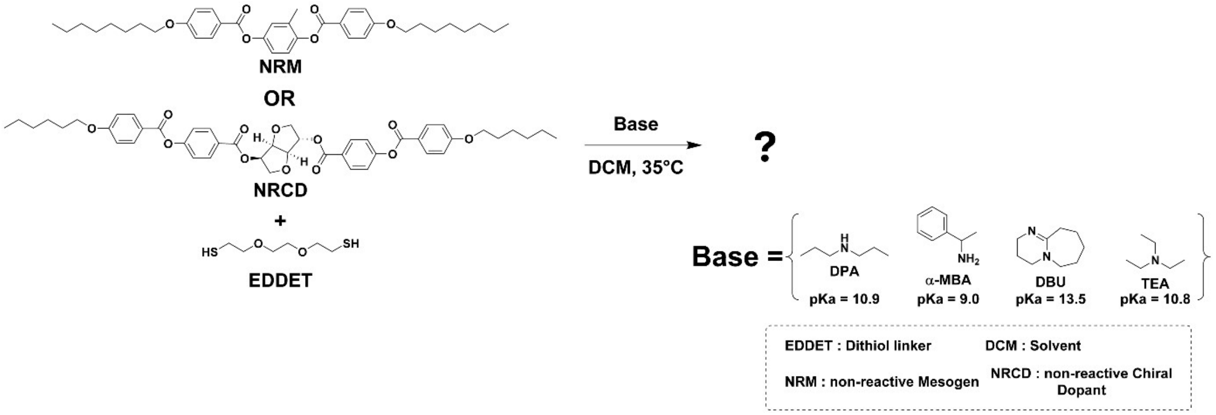

Figure S31 Schematic representation of the possible reaction between non-reactive mesogen (NRM) or non-reactive chiral dopant (NRCD) in the presence of the base and with or without the addition of the dithiol (EDDET) molecule.

The NRM-EDDET mixture with DBU as a base showed additional peaks after 24 h which slightly increased intensity after 48 h. The appearance of a new singlet at 11.7 ppm (**Figure S32**), indicates the presence of a carboxylic acid[3] functional group. Additionally, the broad new signal at 1.7 ppm may correspond to an alcohol group. Together these signals indicate the formation of ester cleavage products. For NRCD, in the absence of the EDDET, additional peaks appeared after 6 h: three distinctive doublets around 8.0 ppm, 6.9 ppm and 6.8 ppm, a quartet that shifted from 6.6 ppm to 6.8 ppm, and a multiplet around 7.8 ppm. (**Figure S33**). When EDDET was added, two relatively strong multiplet peaks appeared around 6.9 ppm and 7.9 ppm within the next two hours (**Figure S34**), which may indicate the presence of a cleaved fragment reacted with EDDET. [4,5] We also observed additional signals between 4.6 and 5.5 ppm, which may be attributed to chemical changes in the isosorbide core. When DPA was introduced as a base into the NRCD mixture without EDDET, additional peaks similar to those observed with DBU appeared after 24 h. However, their intensities remained relatively low even after 48 h (**Figure S35**). When EDDET was present in the mixture, new peaks appeared around 6.9 ppm and 7.9 ppm in the ^{1}H-NMR spectrum (**Figure S36**). These findings indicate that a similar ester cleaving reaction occurs in the presence of DPA. Similarly, for the α-MBA, EDDET, NRCD mixture the same additional peaks were observed (**Figure S37**). The relative peak intensities were even lower and the isosorbide peaks remain unaffected after 48 h (**Figure S38**).

Non-reactive mesogen (NRM) stability experiment in the presence of DBU and EDDET

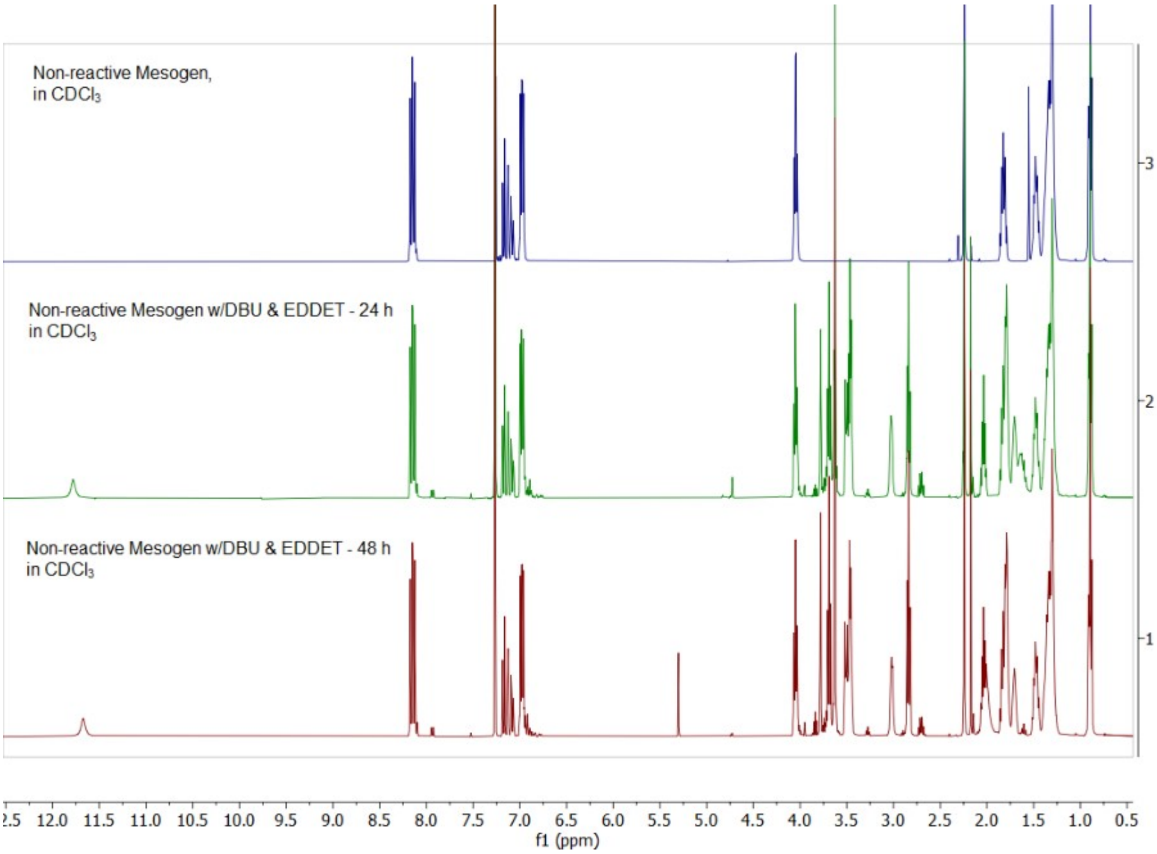

Figure S32 ^{1}H-NMR spectra of NRM in the presence of DBU and EDDET. A distinct peak at 11.68 ppm is visible after 24 hours, that could indicate a presence of a carboxylic acid.

Non-reactive chiral dopant (NRCD) stability experiment in the presence of DBU

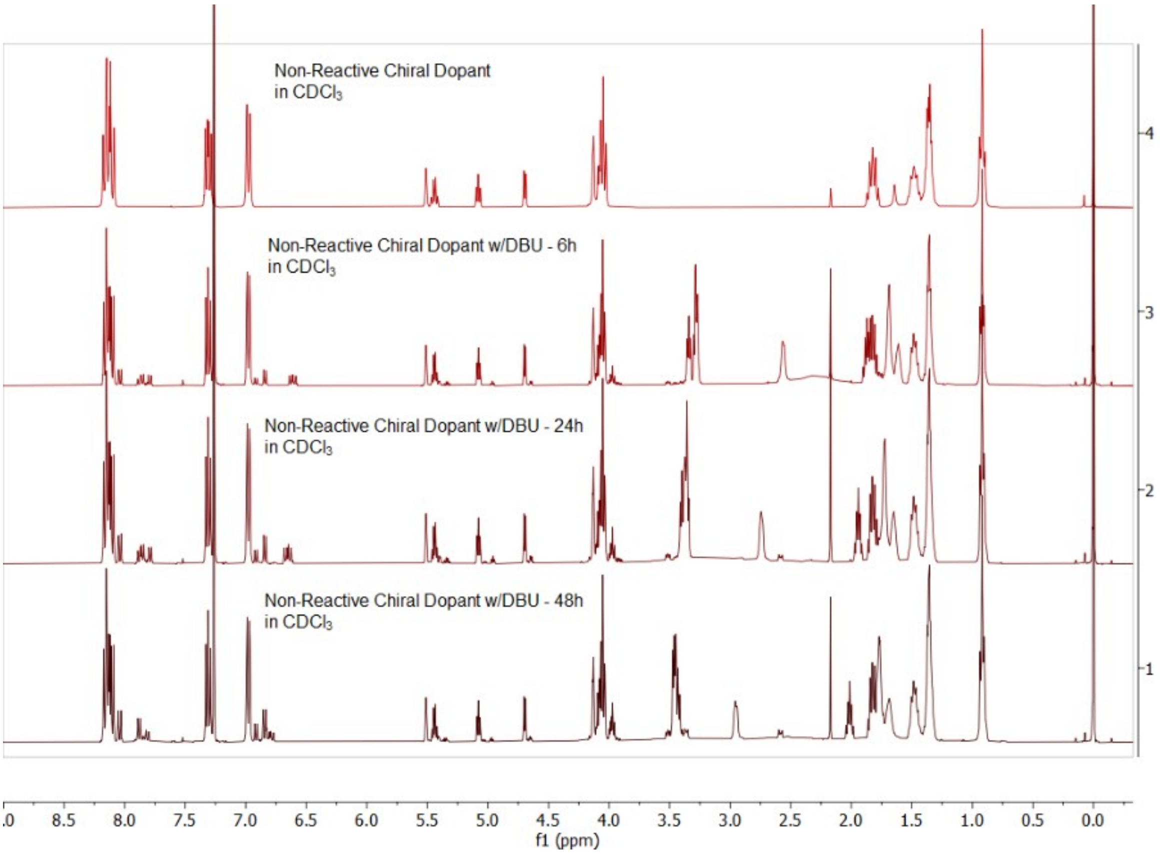

Figure S33. ^{1}H-NMR spectra of NRCD in the presence of DBU. Distinct peaks at 8.04, 6.92, 6.84 and 7.83 ppm are visible after 6 hours.

Non-reactive chiral dopant (NRCD) stability experiment in the presence of DBU and EDDET

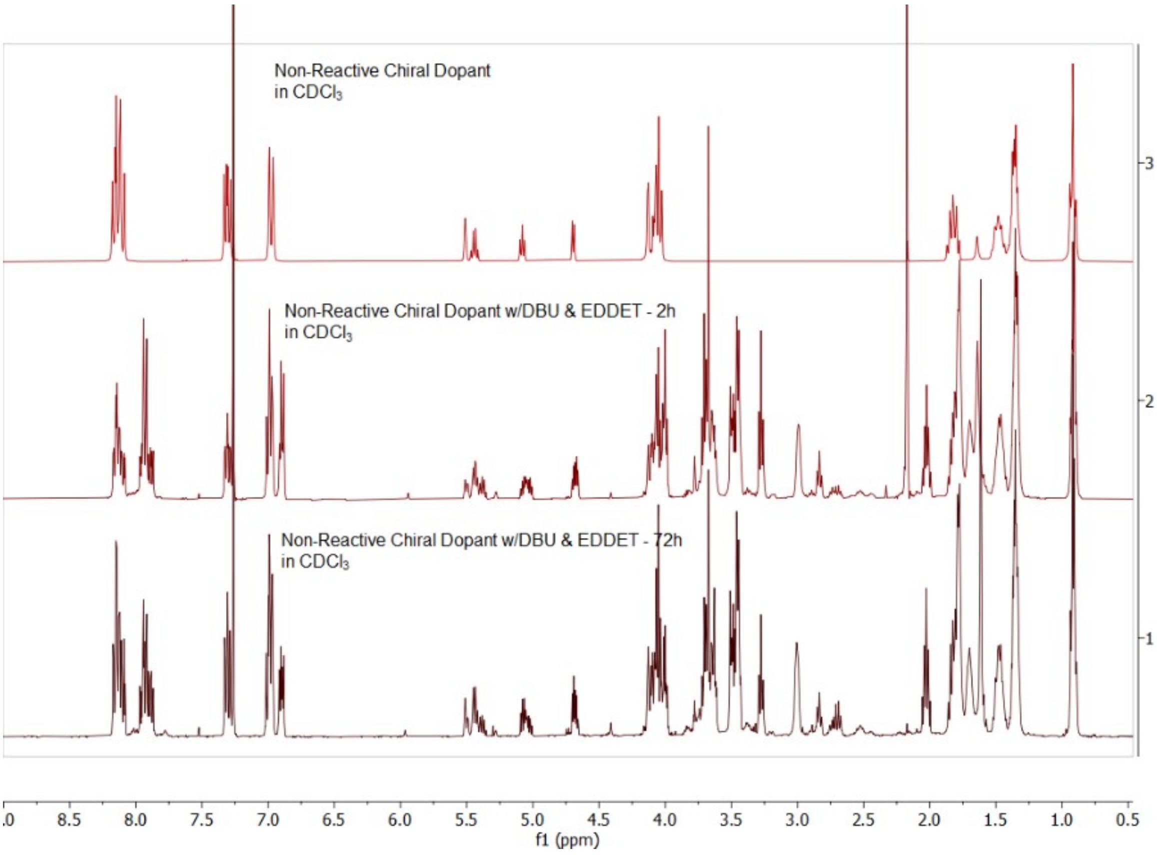

Figure S34 ^{1}H-NMR spectra of NRCD in the presence of DBU and EDDET. Two new distinct peaks at 6.89, and 7.93 ppm are visible after 2 hours.

Non-reactive chiral dopant (NRCD) stability experiment in the presence of DPA

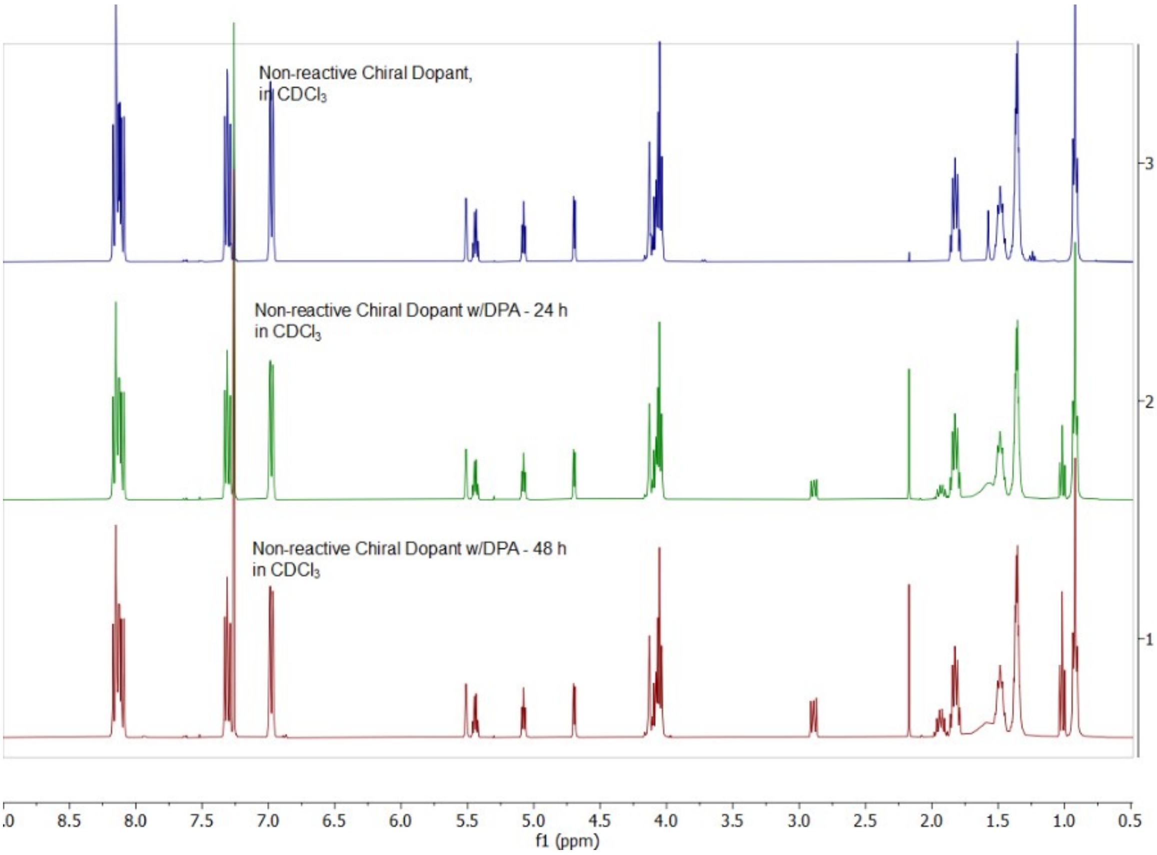

Figure S35. ^{1}H-NMR spectra of NRCD in the presence of DPA

Non-reactive chiral dopant (NRCD) stability experiment in the presence of DPA & EDDET

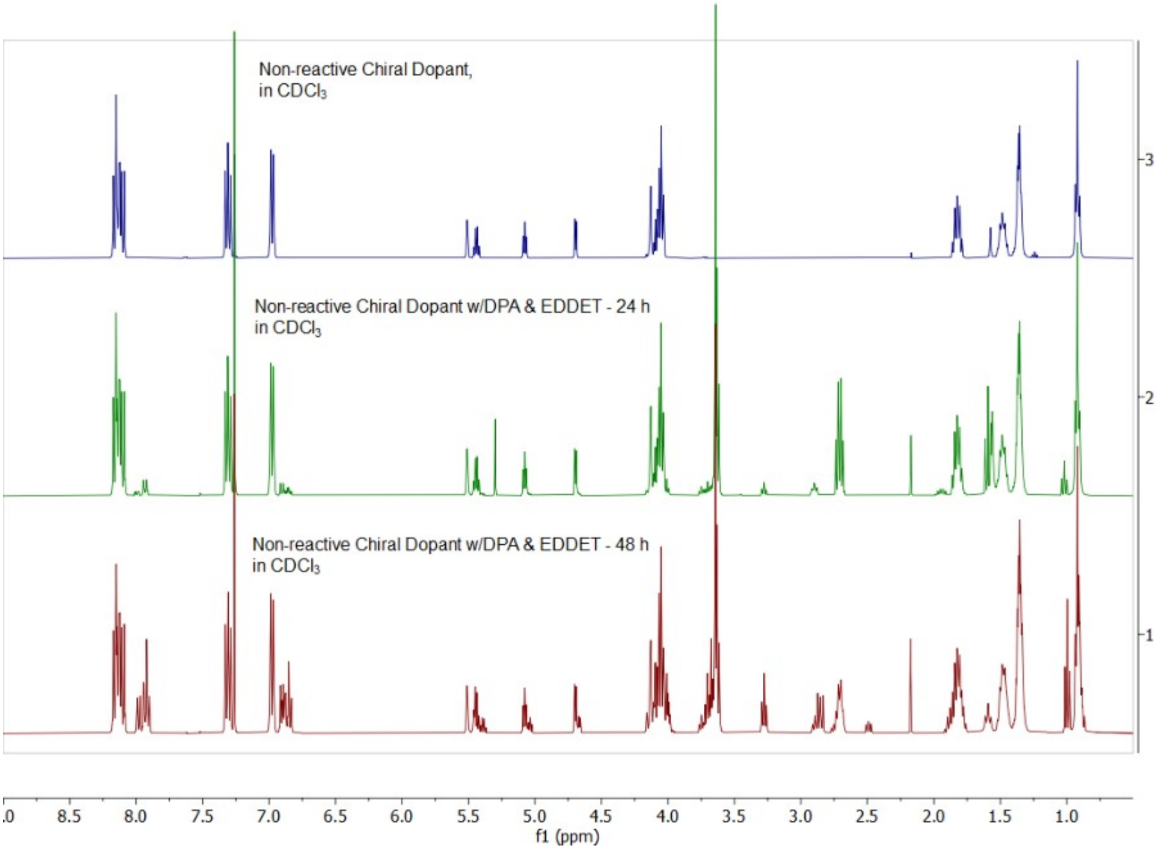

Figure S36 ^{1}H-NMR spectra of NRCD in the presence of DPA and EDDET. Two new distinct peaks at 6.89, and 7.93 ppm are visible after 24 hours.

Non-reactive chiral dopant (NRCD) stability experiment in the presence of α-MBA

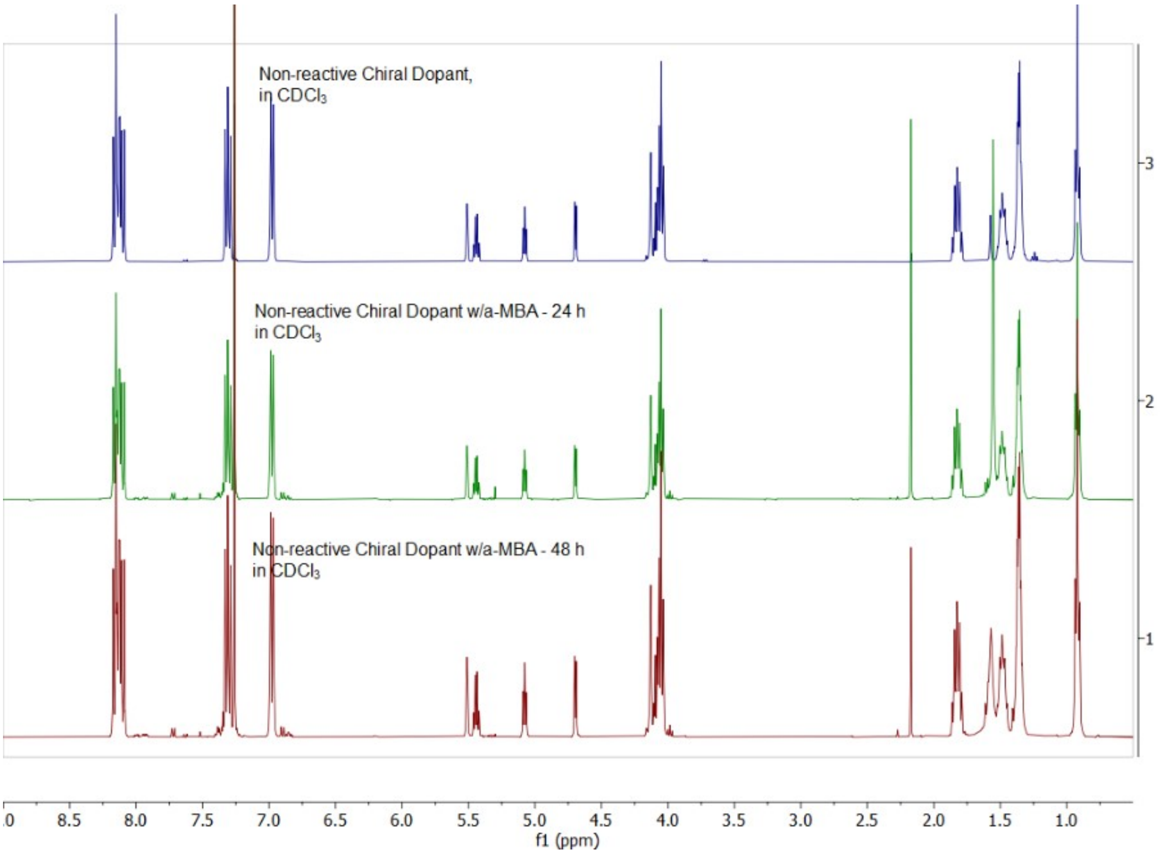

Figure S37 ^{1}H-NMR spectra of NRCD in the presence of α-MBA.

Non-reactive chiral dopant (NRCD) stability experiment in the presence of α-MBA and EDDET

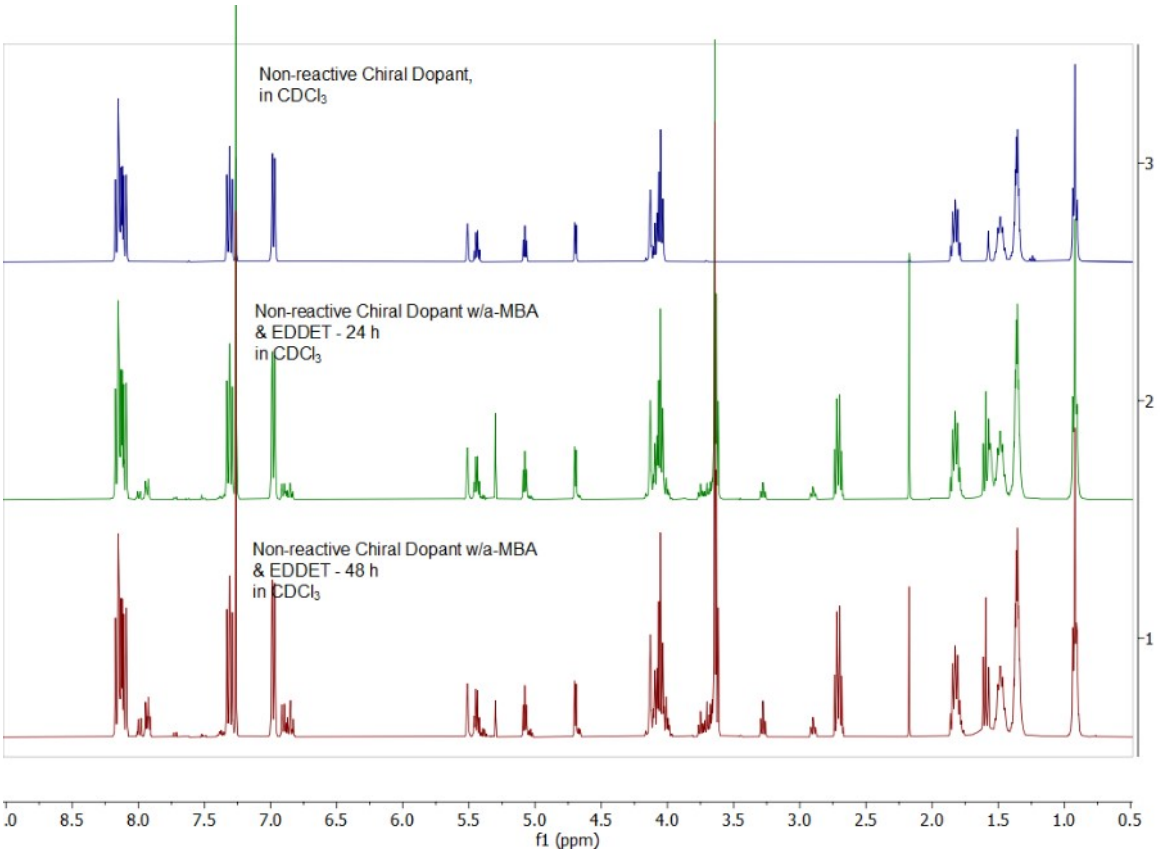

Figure S38 ^{1}H-NMR spectra of NRCD in the presence of α-MBA and EDDET. Two new distinct peaks at 6.89, and 7.93 ppm are visible after 24 hours.

Different solvents and atmospheric conditions to increase the conversion of the TEA catalysed thiol-Michael reaction

When DCM was used as the solvent, with all other reaction conditions kept the same, the conversion of the acrylates in air reached 60% after 24 h, while the reaction under argon showed higher conversion of 77%. In the case of acetone, the acrylate conversion reached 59% under air after 24 h, whereas the reaction under argon displayed a higher conversion of approximately 68%. Remarkably in DMF acrylate conversion was already 82% before the addition of the TEA. After TEA was added, the conversion continued to increase reaching 93% after 24 h. No significant influence of the atmosphere on the acrylate conversion was observed

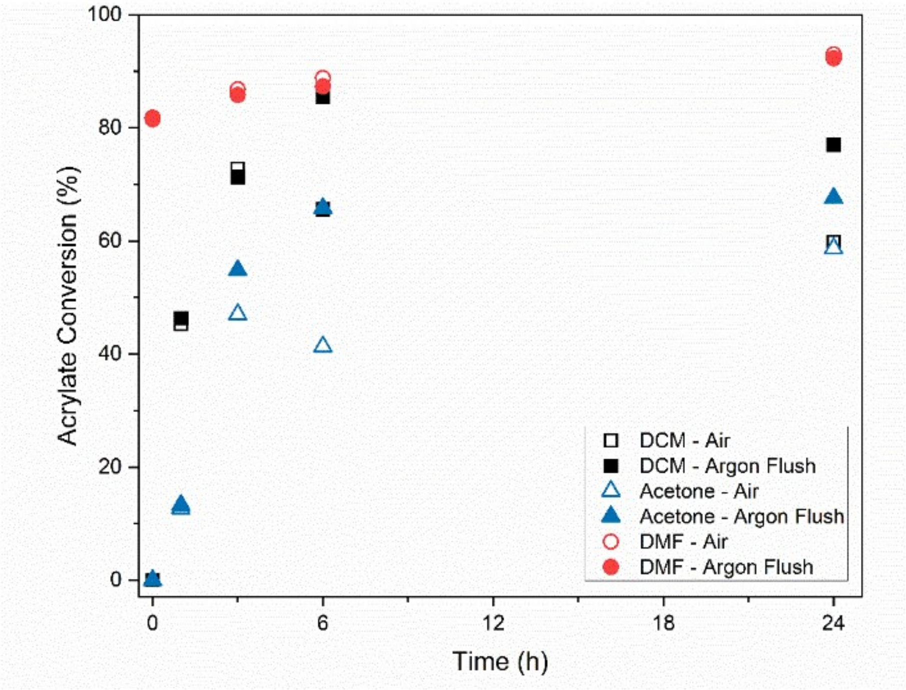

Figure S39 Acrylate conversion via TEA catalysed thiol-Michael addition in different solvents and atmospheric conditions.

in DMF.

TEA catalysed thiol-Michael reaction, 10 mol.% of TEA in 0.5M concentrated solution

In order to increase the yield of the TEA catalysed reaction without compromising the selectivity, the overall concentration of the reactants were doubled to approximately 0.5 M by simply halving the solvent (DCM) volume and the TEA amount was increased to 10 mol.% of the reactants and the reaction kept at 35°C for 24 hours. After the reaction, the solvent was evaporated.

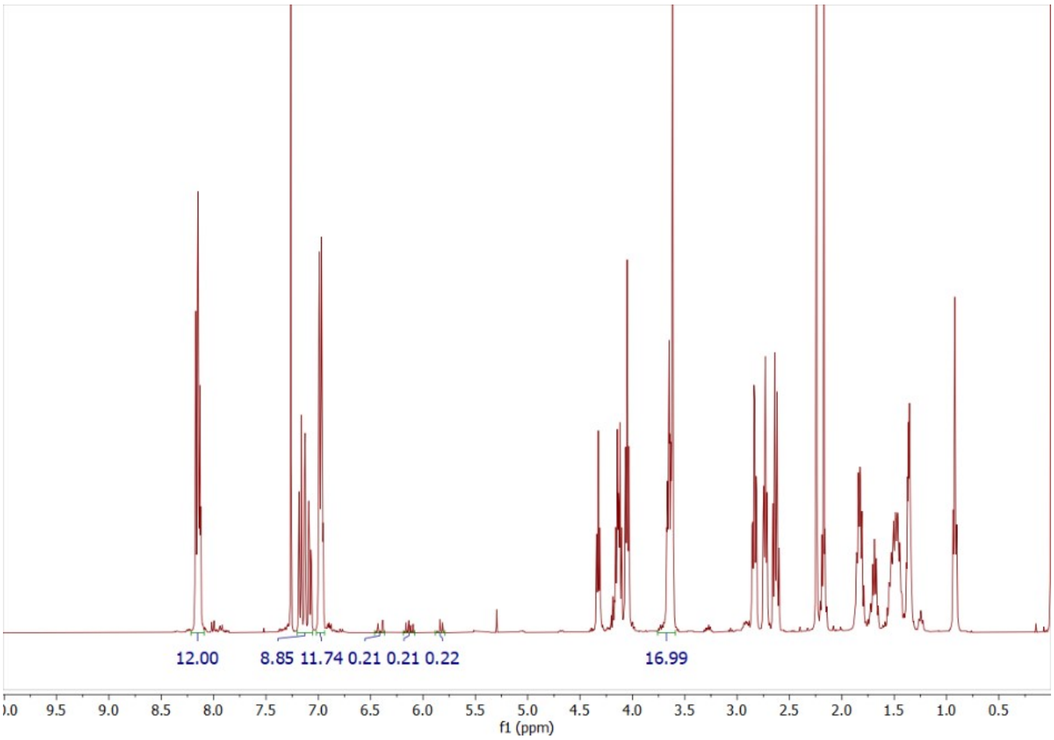

Figure S40. ^{1}H-NMR spectrum of LCO_3 oligomer synthesis catalysed by TEA (10 mol.%) after 24 hours of reaction at 35°C and evaporation of solvent at room temperature overnight.

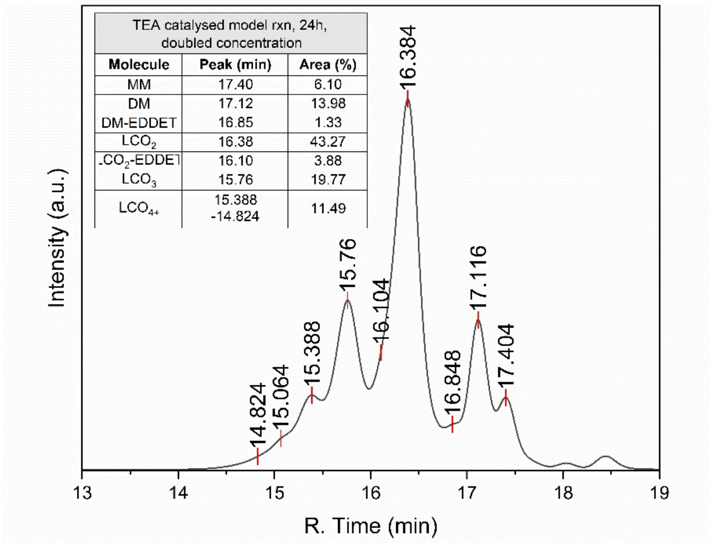

Figure 41 GPC profile of TEA catalysed reaction at 24th hour. The TEA amount is 10 mol% of the reactants and the overall concentration is doubled to 0.5 M by halving solvent volume.

Liquid crystal elastomer (LCE) fabrication from LCOs

LCEs (LCE_{DPA}, LCE_{TEA1}, LCE_{TEA2}) were prepared using DM, EDDET and DPA (6 mol.%) or TEA (6 mol.% or 10 mol.% with doubled concentration) as base DM was put in a 20 mL amber vial. In a separate vial, 229.5 mg (2/3 mol eq.) EDDET was dissolved in 2.5 mL dichloromethane (DCM), vortexed for 30 seconds and transferred into the amber vial via a glass pipette. The vial then washed out with additional 2 mL DCM and vortexed 30 seconds again. The contents was transferred into the amber vial too, achieving 4.5 mL solvent volume in total. The 20 mL amber vial was heated to 35°C by a hot plate while stirred at 300 RPM for 5 minutes. Then, the base catalyst was added to the vial at t_0 = 0 h. After addition of the catalyst, the amber vial was gently purged with argon for 30 s and then mechanically closed with a PTFE lined screw cap. The reactions were continued up to 18 hours. After oligomerisation Irgacure 819 was added as photoinitiator (1wt.% of the LCO mass) to each batch. After mixing for 30 minutes, the oligomer mixtures are casted onto glass which was cleaned with acetone and IPA and dried with air before use. The solvent was slowly evaporated at room temperature overnight. Subsequently films were exposed to an EXFO omnicure S2000 UV lamp, set to 30 mW/cm^2, under nitrogen atmosphere. Top and bottom of the surfaces were exposed to UV irradiance for 15 minutes each.

The degree of polymerisation (DP) showed values of 3.44, 2.20, and 3.05, respectively (LCO_{DPA}, LCO_{TEA1}, and LCO_{TEA2}, **Figure S42, Figure S43**, and **Figure S44**). [6]

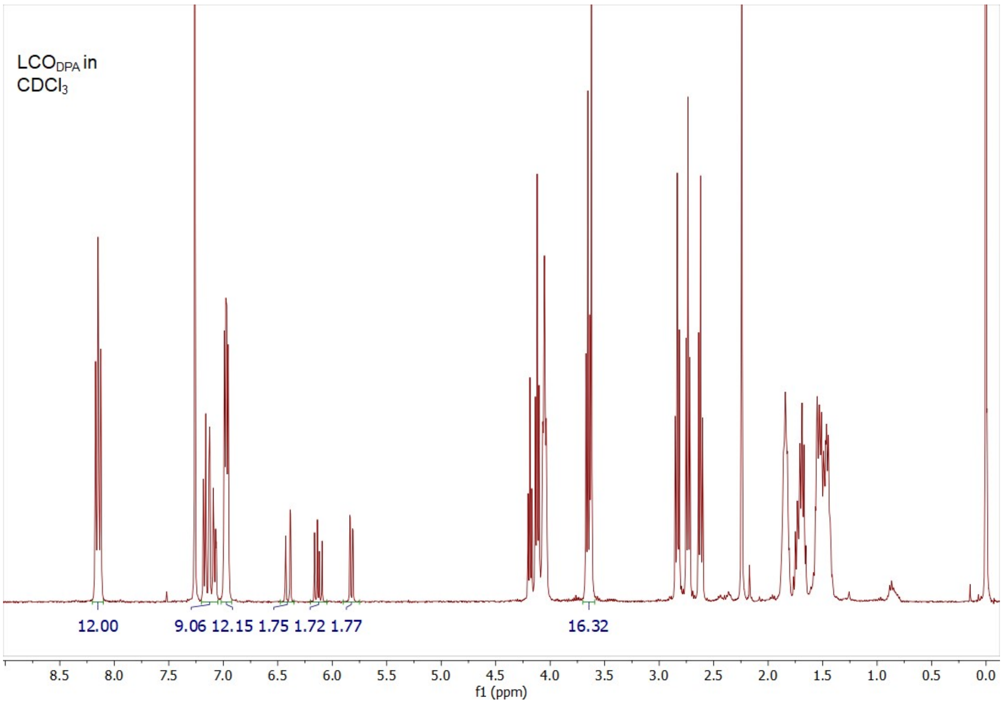

Figure S42 ^{1}H-NMR spectrum of the oligomer mixture catalysed by DPA (6 mol.%) after 18 hours of reaction at 35°C and evaporation of solvent at room temperature overnight.

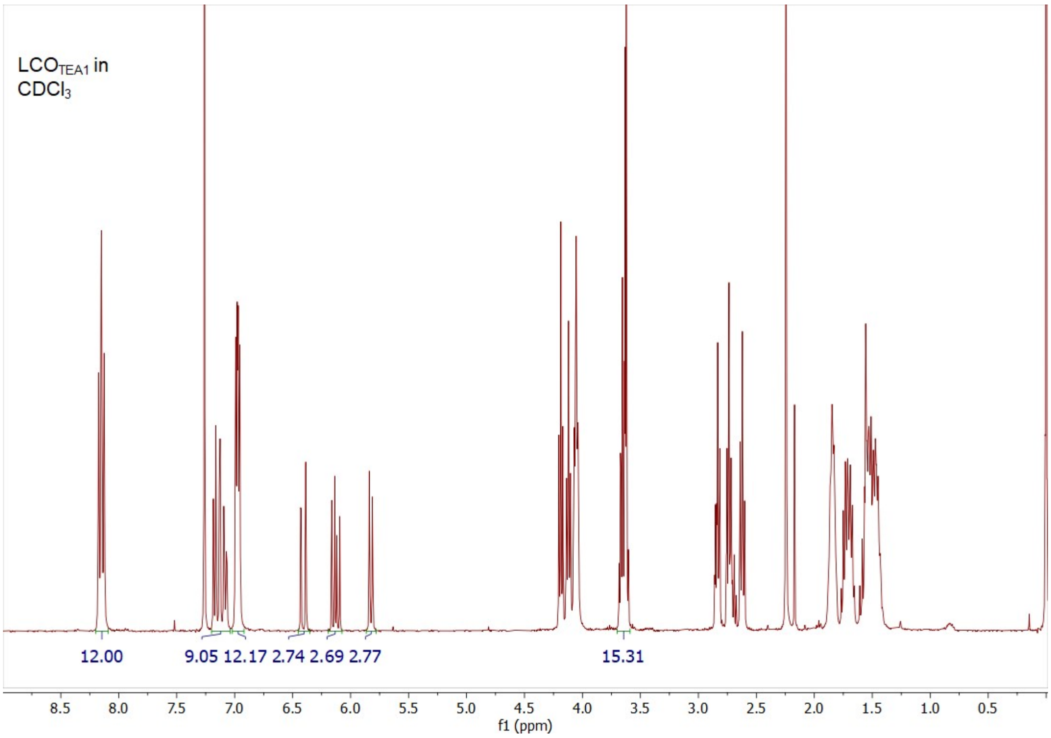

Figure S43 ^{1}H-NMR spectrum of oligomer mixture catalysed by TEA (6 mol.%) after 18 hours of reaction at 35°C and evaporation of solvent at room temperature overnight.

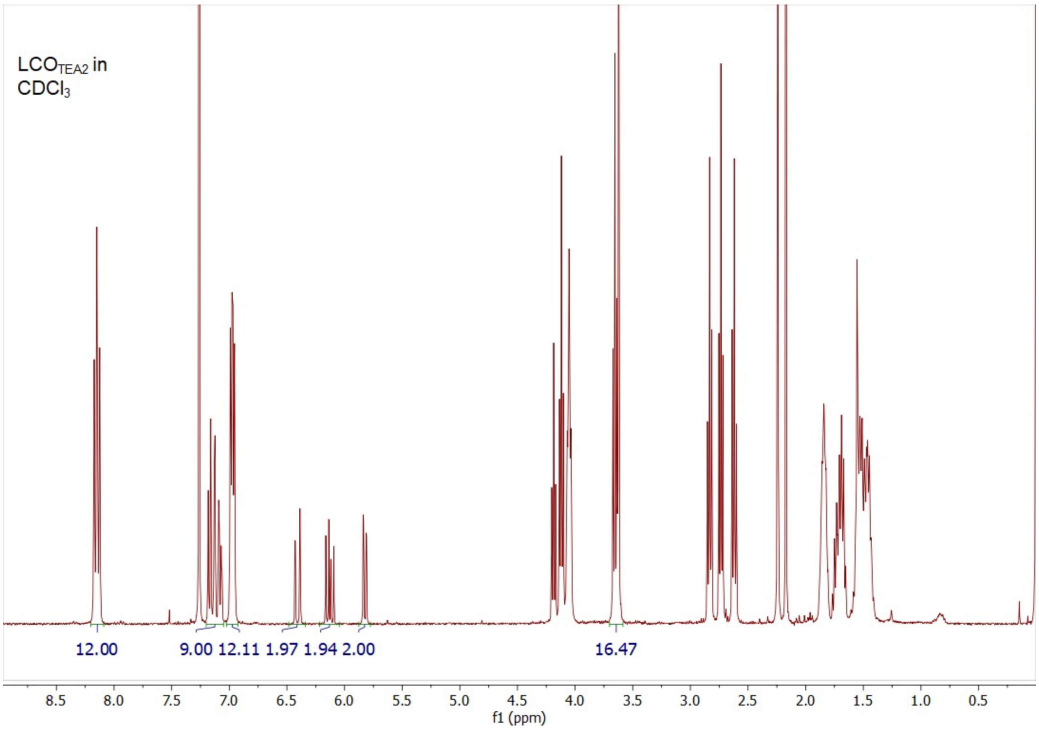

Figure S44 ^{1}H-NMR spectrum of oligomer mixture catalysed by TEA (10 mol.%, doubled reactant concentration), after 18 hours of reaction at 35°C and evaporation of solvent at room temperature overnight.

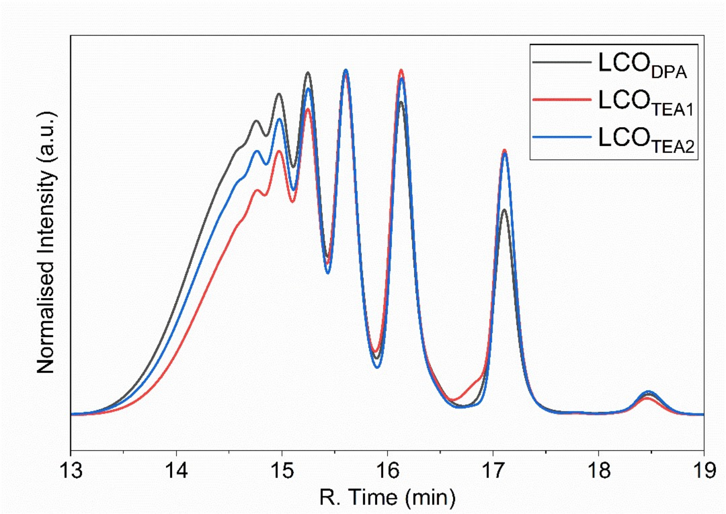

Figure S45 GPC profiles of the oligomer mixtures (LCO$_{DPA}$, LCO$_{TEA1}$, and LCO$_{TEA2}$). [6]

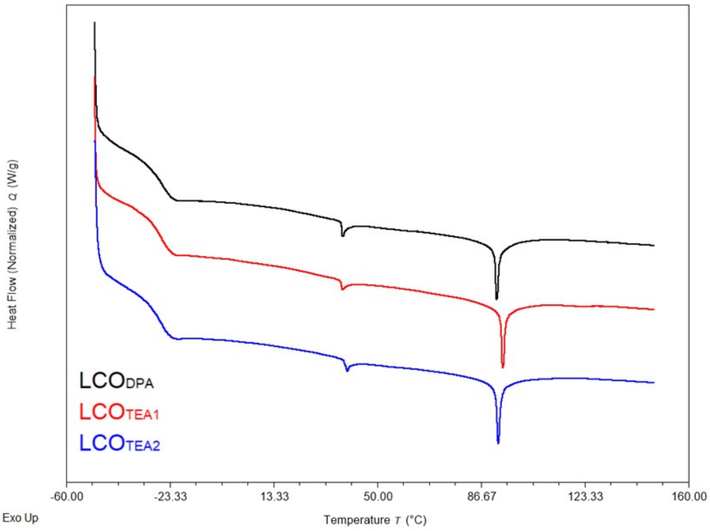

Figure S46 DSCs of LCO$_{DPA}$, LCO$_{TEA1}$, and LCO$_{TEA2}$

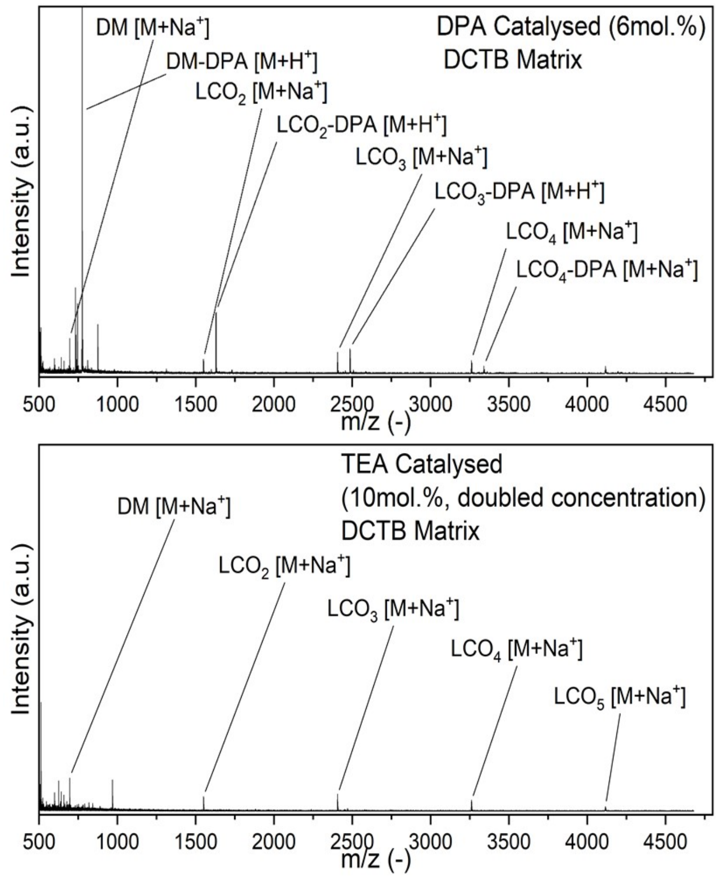

Figure S47 MALDI-ToF spectra of LCO_{DPA} and LCO_{TEA2}

The dynamic mechanical thermal analyses (DMTA) of the LCEs were done by cutting 5 mm wide, 20 mm long, and approximately 350 µm thick samples. Firstly, The LCE samples were tested for analysing storage and loss modulus, and tanδ between -50°C and 150°C with a heat ramp of 5°C/min. The samples showed storage modulus of 48.9 MPa, 34.5 MPa, and 32.4 MPa for LCE_{TEA2}, LCE_{DPA}, and LCE_{TEA1}, respectively at 20°C. As the referee predicted, the more crosslinked TEA sample LCE_{TEA2}, showed the highest storage modulus. The tanδ values at the same temperature were found as 0.38, 0.35, and 0.30 for LCE_{TEA2}, LCE_{DPA}, and LCE_{TEA1}, respectively.

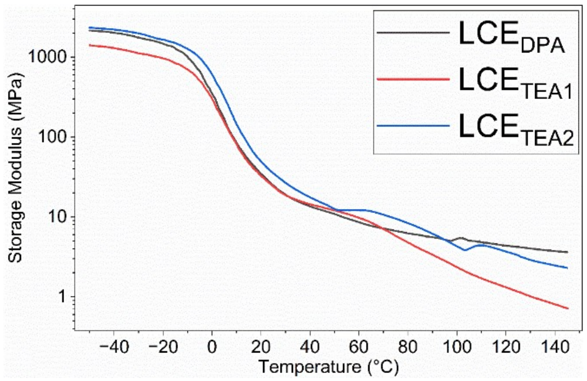

Figure S48 Storage modulus analyses of LCE_{DPA}, LCE_{TEA1}, and LCE_{TEA2}.

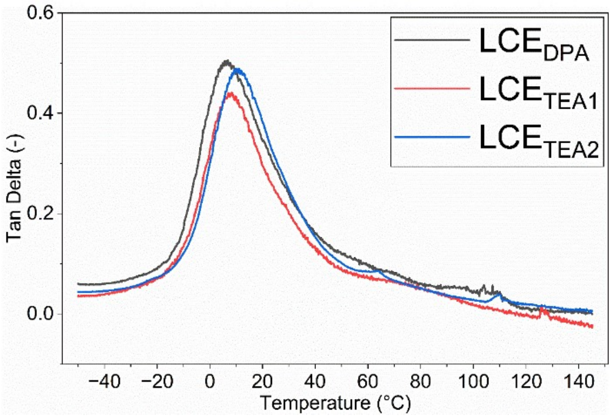

Figure S49 Tanδ analyses of LCE_{DPA}, LCE_{TEA1}, and LCE_{TEA2}.

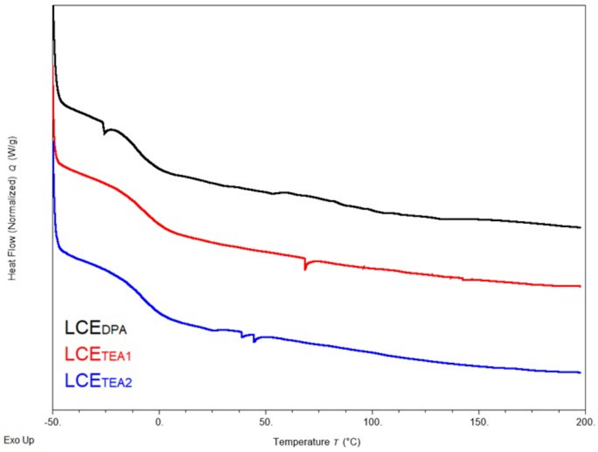

Figure S50 DSCs of $LCE_{DPA,}$ $LCE_{TEA1,}$ and LCE_{TEA2}

References

1 H. Sentjens, J. M. A. Bloemers, J. Lub, C. L. Gonzalez, A. J. J. Kragt and A. P. H. J. Schenning, *Liq. Cryst.*, 2024, **51**, 1651–1663.

2 S. L. Arora, J. L. Fergason and T. R. Taylor, *Journal of Organic Chemistry*, 1970, **35**, 4055–4058.

3 B. Singh, A. Pandey and S. K. Singh, *Molecular Crystals and Liquid Crystals*, 2010, **517**, 127–137.

4 X. Qi, Z. P. Bao and X. F. Wu, *Organic Chemistry Frontiers*, 2020, **7**, 885–889.

5 P. Gopinath, R. S. Vidyarini and S. Chandrasekaran, *European J. Org. Chem.*, 2009, 6043–6047.

6 H. Sentjens, A. J. J. Kragt, J. Lub, M. D. T. Claessen, V. E. Buurman, J. Schreppers, H. A. Gongriep and A. P. H. J. Schenning, *Macromolecules*, 2023, **56**, 59–68.
